# Solid solution quantification from full powder X-ray diffraction profile: novel application of multivariate calibration

**DOI:** 10.1107/S1600576725006910

**Published:** 2025-09-04

**Authors:** Laura Macchietti, Nicholas Kassouf, Giovanni Valenti, Dora Melucci, Fabrizia Grepioni

**Affiliations:** a Department of Chemistry ‘Giacomo Ciamician’ University of Bologna Via Gobetti 85 40129Bologna Italy; Shiv Nadar Institution of Eminence, India

**Keywords:** multivariate analysis, powder X-ray diffraction, quantitative models, partial least-squares regression, solid solutions

## Abstract

Non-stochiometric crystals, such as solid solutions, represent a valid strategy for fine-tuning the properties of materials from inorganic pigments to active pharma­ceutical ingredients. The change is achieved by substitution of structurally similar compounds within the crystal structure, resulting in a minor variation of cell parameters, and it can be experimentally observed as a shift of the X-ray diffraction profile, with the shift depending on the substitutional amount. After proper alignment of the diffraction data, chemometric methods can detect the evolution of the solid solution profile and correlate it to the molar composition, creating rapid quantitative models. The solid solutions chosen for this work are NA_2_·FA_*x*_SA_1−*x*_ and IN_2_·FA_*x*_SA_1−*x*_, *i.e.* co-crystals of nicotin­amide (NA) and isonicotinamide (IN) with fumaric acid (FA) and succinic acid (SA) in different proportions; they are used as model systems for the development of principal component regression and partial least-squares models for the quantification of solid solution composition. Different alignment strategies are presented, and the results obtained from test samples show the prediction efficacy of the proposed approach.

## Abstract 2

A new application of quantitative chemometric models (principal component regression and partial least-squares regression) to powder X-ray diffraction data is tested. The approach allows evaluation of the relative composition of solid solutions *via* the characteristic profile shift. A method relying on Bragg–Brentano geometry measurements is presented, along with different alignment strategies to treat experimental error due to sample displacement.

## 1. Introduction

The quest for methods that allow the modification, improvement or fine-tuning of the solid-state properties of molecular crystals, without changing the chemical nature of the molecule of interest, as in the case of active pharmaceutical ingredients (APIs), has produced in the past few decades an astonishing and still growing number of new and varied crystal forms, mainly and broadly grouped as polymorphs, molecular or ionic co-crystals, and solid solutions (Braga *et al.*, 2022 ▸; Braga, 2023 ▸; Grepioni *et al.*, 2022 ▸). Both co-crystals and solid solutions are formed via co-crystallization of two or more compounds that are solid under ambient conditions in their pure form but can be mixed in a reproducible way via exploitation of stabilizing noncovalent interactions (hydrogen and halogen bonds, π–π stacking, ion–dipole *etc.*) (Haque *et al.*, 2023 ▸). The fundamental difference between co-crystals and solid solutions, however, is in the stoichiometric ratio of the components: while it is fixed in co-crystals, it can be varied in solid solutions, either *in continuum* or within defined compositional ranges.

Co-crystal formation can be controlled through rational selection of co-formers and crystallization conditions, offering new opportunities to modulate the properties of APIs while maintaining their therapeutic activity (Wouters *et al.*, 2018 ▸). Improvements have been reported in terms of stability, solubility, bioavailability, permeability and mechanical properties, with no alteration of the pharmacological properties (Narala *et al.*, 2021 ▸; Duggirala *et al.*, 2016 ▸).

In the pharmaceutical field, solid solutions [or mixed crystals, as described in the seminal work of Kitaigorodsky (1984 ▸)] enable modification of drug dissolution and absorption rates (Lusi, 2018 ▸). Improvement of these properties could lead to increased drug efficacy. Improvements in thermal and mechanical properties that are crucial in tablet formation and storage are also reported (Spoletti *et al.*, 2023 ▸). The ability to modify continuously characteristics such as solubility, dissolution rate and thermal stability makes solid solutions particularly interesting for optimizing pharmaceutical formulations (Lusi *et al.*, 2015 ▸; Shemchuk *et al.*, 2016 ▸; Cruz-Cabeza *et al.*, 2018 ▸; Fonseca *et al.*, 2018 ▸; Romasanta *et al.*, 2017 ▸; Dabros *et al.*, 2007 ▸). Crystal engineering of solid solutions also offers opportunities for tailoring properties of pigments and luminescent materials (Zhen *et al.*, 2015 ▸; Newsome *et al.*, 2019 ▸; Xu *et al.*, 2022 ▸; Xiong *et al.*, 2018 ▸).

In the present work we have investigated the behaviour of co-crystal solid solutions, which represent a particular case of solid solutions where a third component is added that acts as a ‘solid solvent’ (Lusi *et al.*, 2015 ▸; Cruz-Cabeza *et al.*, 2018 ▸; Bruni, 1900 ▸). The third component can act as a stoichiometric coformer, while the two other components mutually substitute each other (Oliveira *et al.*, 2008 ▸; Srisanga & ter Horst, 2010 ▸; Chen *et al.*, 2010 ▸; Roex *et al.*, 2007 ▸; Hasell *et al.*, 2012 ▸). The combination of co-crystal and solid solution properties can represent a further improvement and fine-tuning in altering the properties of the molecule of interest.

Since controlling and characterizing crystalline solid forms is essential for obtaining materials with the desired properties, not only qualitative identification of different solid forms but also their quantification is needed. This aspect is crucial for quality control and regulatory compliance, especially in the pharmaceutical field, where solid formulations represent the most popular product (Shaikh *et al.*, 2018 ▸). Therefore, the search for new methods to quantify solid forms is an active field of research, with multiple techniques employed from X-ray diffraction (XRD) (Zappi *et al.*, 2019 ▸) and spectroscopy (Kassouf *et al.*, 2024 ▸) to thermal analysis and solid-state NMR.

Tao *et al.* (2025 ▸) reviewed the newest quantitative method­ologies, primarily focused on phase quantification of different components inside a mixture. However, in the presence of solid solutions, changes in composition happen within a single crystallographic structure and, due to the modulation of physicochemical properties, determination of the relative composition of the material can be of interest. Powder X-ray diffraction (PXRD) represents a particularly relevant methodology when dealing with continuous systems such as solid solutions, where the similar chemical properties of the constituent elements could make traditional analytical methods ineffective. The theoretical foundation of this application is based on the possibility of detecting lattice parameter variations caused by the substitution of one component for a second one in the crystalline unit cell. Vegard’s law (Denton & Ashcroft, 1991 ▸) establishes a linear relationship between lattice parameters and composition, expressed as a molar fraction, with changes in the lattice parameters causing peak shifts in the diffraction profiles (d’Agostino *et al.*, 2018 ▸). Although the progression observed experimentally can diverge from ideal linearity, this law provides a reliable framework for quantitative determinations based on PXRD, as shown in cases for which conventional analytical techniques could not be successfully employed to discriminate between constituent species (Cherin, 1973 ▸).

Rietveld and Pawley refinements, within the traditional PXRD approach of pattern-fitting methods, provide detailed structural information, including lattice parameters, and can be used for the characterization of solid solutions, tracing the changes in the crystal structure (Hurle *et al.*, 2022 ▸; Kobayashi *et al.*, 2024 ▸). Only a limited number of examples, however, have been reported in the literature for quantification of solid solution composition by these methods (Vogrin *et al.*, 2019 ▸), and they also rely on the availability of known crystal structures for all the phases involved.

Multivariate analysis (MA) is becoming a strategic tool for quantitative determinations (Tao *et al.*, 2025 ▸) and it is finding application to diffraction data, constituting a powerful complementary tool for crystalline materials characterization (Lopresti *et al.*, 2023 ▸; Macchietti *et al.*, 2024 ▸; Yuan *et al.*, 2024 ▸). Dedicated software is also available, combining XRD data treatment and MA calculations (Barr *et al.*, 2004 ▸; Caliandro & Belviso, 2014 ▸). Principal component analysis (PCA) and partial least-squares (PLS) regression can be employed to transform complex data sets into meaningful information: PCA enables visualization of complex data structures by reducing dimensionality while preserving essential variance information, facilitating pattern recognition and outlier detection in crystalline samples; PLS regression establishes quantitative relationships between analytical data and phase composition, allowing for accurate determination of crystalline phases even in complex mixtures (Rajalahti & Kvalheim, 2011 ▸). Advantages of the MA approach include the possibility of data analysis in the absence of crystallographic reference data or familiarity with profile-fitting techniques, as well as providing automated calculations suitable for big data analysis or further integration into multi-step algorithms.

**Figure 1:**
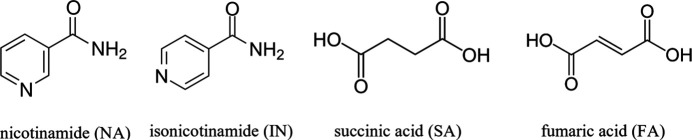
Molecular structures of the compounds used for the solid solution model systems.

Within this framework we propose a quantitative multivariate approach to evaluate solid solution compositions from experimental PXRD profiles, employing principal component regression (PCR) and PLS to enhance the linear correlation depicted by Vegard’s law. To this end, we re-prepared via mechanochemistry the known co-crystal solid solution of isonicotinamide (IN) with fumaric acid (FA) and succinic acid (SA), IN_2_·FA_*x*_SA_1−*x*_ (**IN-SS** in the following, Fig. 1 ▸) (Oliveira *et al.*, 2008 ▸), selected as a test model for our quantification procedure. As a second step, we synthesized and analysed the analogous new co-crystalline system with nicotinamide (NA), NA_2_·FA_*x*_SA_1−*x*_ (**NA-SS** in the following, Fig. 1 ▸), which turned out to be different from the isonicotinamide structure, as in this case only partial miscibility of SA and FA was observed. The solid solution behaviour was investigated and characterized by PXRD, Pawley refinement and differential scanning calorimetry (DSC). Our results show how chemometrics, combined with PXRD data and an appropriate alignment strategy, can provide robust quantitative models and help in highlighting features of solid solution profiles.

## 2. Experimental

### 2.1. Materials

NA, IN, SA and FA were purchased from Sigma–Aldrich and used without further purification.

### 2.2. Solid solution syntheses

The known **IN-SS** co-crystal, with formula IN_2_·FA_*x*_SA_1−*x*_ (Oliveira *et al.*, 2008 ▸), shows complete solubility of FA and SA in the whole compositional range (0 < *x* < 1). Samples for model training were prepared with FA molar fractions (referred to only as fraction hereinafter) in the range 0−1, in 0.2 increments, while control samples for model validation were prepared with FA fractions of 0.3, 0.5 and 0.7. All products were obtained via mechanochemical synthesis by liquid-assisted grinding (LAG) (Ying *et al.*, 2021 ▸). IN, FA and SA were weighed at the correct molar ratio to a total weight of 200 mg and loaded inside a 5 ml ball-milling steel jar with two steel spheres of 5 mm. Ethanol (50 µl) was added to facilitate the conversion. A Retsch MM 200 ball-milling apparatus was used, operating at 25 Hz for 30 min.

Analogous solid solutions of formula NA_2_·FA_*x*_SA_1−*x*_, **NA-SS**, were also prepared, with NA replacing IN. As partial solubility of FA and SA was observed, the FA fraction (*x*) was varied only in the 0−0.6 compositional range. Samples for the quantitative model were obtained via LAG, with a procedure similar to that used for the **IN-SS** co-crystals, but with the milling time increased to 60 min to ensure consistent completeness. Due to the limited miscibility, model validation was tested only for the NA_2_·FA_0.3_SA_0.7_ and NA_2_·FA_0.5_SA_0.5_ compositions.

### 2.3. NA-SS characterization

The formation of solid solutions for the NA co-crystals was verified by thermal analysis and unit-cell parameter calculations via Pawley refinement. DSC was performed with a TA Instruments Q10 apparatus fitted with a standard DSC cell, equipped with a Discovery Refrigerated Cooling System (RCS90, TA Instruments) under a nitro­gen atmosphere (purge flow 20 ml min^−1^). The samples (3–8 mg) were placed in aluminium pans. All samples were cooled to 0°C and then heated to 170°C. Pawley refinement was performed with the software *TOPAS* (Version 5; Coelho, 2018 ▸). A Chebyshev function with two parameters was used to fit the background and a pseudo-Voigt function was used for the peak profile.

### 2.4. PXRD

Diffractograms used for Pawley refinement were recorded in Bragg–Brentano geometry for each selected composition of NA_2_·FA_*x*_SA_1−*x*_ (*x* = 0, 0.2, 0.4 and 0.6) on a PANalytical X’Pert Pro automated diffractometer equipped with an XCelerator detector using Cu *K*α radiation (λ = 1.5418 Å) in the 4–60° 2θ range with four scans (continuous scan mode, step size 0.0167°, counting time 40.005 s, Soller slit 0.04 rad, antiscatter slit 1/2, divergence slit 1/4, 40 mA, 40 kV).

Diffractograms used for the manual alignment data set of both **NA-SS** and **IN-SS** systems were recorded on a PAN­alytical X’Pert Pro in the 4–40° 2θ range (continuous scan mode, step size 0.0334°, counting time 50.165 s, Soller slit 0.02 rad, antiscatter slit 1/2, divergence slit 1/4, 40 mA, 40 kV). An internal standard was added to the IN_2_·FA_*x*_SA_1−*x*_ samples to align the pattern correctly and compensate for the 2θ shift due to experimental sample displacement. Before each measurement, finely ground NaCl (20 mg) was mixed with 80 mg of solid solution. In the case of the NA system, the alignment was first tested with the use of an internal standard. After the identification of a low-angle peak, constant for all solid solution compositions, all acquisitions were carried out on clean samples without further addition.

The same **IN-SS** samples, containing NaCl as internal standard, were also measured on a PANalytical Empyrean automated diffractometer using Cu *K*α radiation (λ = 1.5418 Å) in the 4–40° 2θ range. The instrument, operating at 40 kV/40 mA, was equipped with Bragg–Brentano HD optics. A PIXcel3D area detector was used for data collection [continuous scan mode, step size 0.0263°, counting time 46.655 s, incident beam Soller slit 0.03 rad, diffracted beam Soller slit 0.02 rad, divergence slit (incident beam) 1/4, antiscatter slit (diffracted beam) 1/4]. Samples were placed inside a quartz sample holder and aligned with respect to the zero of the goniometer, using the *XYZ* sample stage and its auto-alignment function.

### 2.5. Data analysis

#### 2.5.1. Pretreatment and alignment

##### 2.5.1.1. Manual alignment

Background correction was applied to all diffractograms prior to any other operation. Background correction and manual alignment were performed with the *X’Pert High Score Plus* software (PANanlytical). Diffractogram alignment was carried out using the correct displacement function, since it returns an adjusted pattern with the same 2θ coordinates as the original acquisition, a necessary requirement for the subsequent matrix operations. For each data set one acquisition was selected as a reference pattern. For all subsequent samples the 2θ shift (Δ2θ) of the identified alignment peak was estimated, with respect to the reference sample, through the adjust to anchor option. Δ2θ was then converted into sample displacement (*s*) by equation (1) (Parrish & Langford, 2006 ▸), with *R* being the goniometer radius:
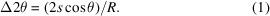
Lastly, the estimated *s* value was inserted into the correct displacement function and an adjusted profile was obtained. The nicotinamide co-crystal acquisitions were aligned with respect to the first low-angle peak at 6.3° 2θ, while the isonicotinamide profiles were aligned with respect to the main NaCl peak at 31.7° 2θ.

##### 2.5.1.2. Automated alignment

Alternatively, pattern pretreatment was integrated into the Python script used for the chemometric calculation (see Section 2.5.3). Background correction was implemented with the SNIP algorithm (Ryan *et al.*, 1988 ▸), while for the alignment a simplified version of the alignment by peak correspondence presented by Guccione *et al.* (2018 ▸) was used. Detection of the relevant peaks was carried out through the find_peaks function (see also Section 2.5.2) from the *SciPy* library (Virtanen *et al.*, 2020 ▸), setting the prominence and width parameters to values that ensured a constant identification of peaks. Reproducibility is needed to match the alignment peak correctly between the reference and *n*th sample during the correspondence step. This second step was simplified, since the 2θ correction needed for solid solution alignment requires only the correspondence of the alignment peak; Δ2θ was calculated as the difference between the positions of the alignment peak in the first and *n*th samples, and then used to shift the 2θ axis of the *n*th sample accordingly. Lastly the corrected profile was interpolated to resample it at the original 2θ values, needed for the chemometric calculation. The methodology used for the definition of the peak position and its impact on the alignment are detailed in the results, Section 3.1.4, and in the supporting information (Section S2).

##### 2.5.1.3. Instrumental alignment

Acquisitions carried out on the Empyrean diffractometer were corrected only for background. Due to the instrumental alignment of the sample-stage position, no further correction of the diffractograms was needed, and the alignment of the reference peak was only verified by visual inspection of the data before construction of the final data set.

#### 2.5.2. Peak position analysis

Statistical analysis of peak positions was carried out on the raw experimental diffractograms, as opposed to a fitted profile, to observe the variability present in the chemometric data. The peak identification algorithm developed for the automated alignment method was employed for this purpose. The find_peaks function was optimized in its parameters to allow the identification of 13 main peaks for nicotinamide. The 2θ value corresponding to the middle point of the peak width, calculated at 80% of the peak height, was taken as the peak position (the peak height percentage will be referred to as level from the baseline, as commonly considered, while the find_peaks function expects the value as a fraction from the peak). The average value of three samples for each solid solution composition and the associated standard deviation were determined.

#### 2.5.3. Multivariate analysis

Data sets were composed of XRD profiles of solid solutions at different SA/FA compositions, corrected for background and aligned if needed (Section 2.5.1). When the internal standard was present in the acquisition the corresponding 2θ range was excluded from the calculation, since the NaCl peak is not relevant for the solid solution progression; a potential intensity variation of the standard could also incorrectly influence the model. The training set contained a total of 12 samples for the **NA-SS** system (three independent syntheses of four increasing FA fractions: 0, 0.2, 0.4 and 0.6) and 18 samples for the **IN-SS** co-crystals (three independent syntheses of six fractions: 0, 0.2, 0.4, 0.6, 0.8 and 1). The expected FA fraction was used as a response to the regression model.

Before any matrix operation, the XRD profiles were normalized to remove the variability in intensity associated with sample preparation. Area (Caliandro *et al.*, 2013 ▸) or maximum (Urbano-Cuadrado *et al.*, 2008 ▸) normalizations were tested.

PCA (Blanco Romía & Alcalà Bernàrdez, 2009 ▸) was used for a preliminary exploration of all data sets to verify the presence of sample clustering on the basis of the FA content. If separation was satisfactory, a quantitative regression model was developed. For the **NA-SS** set, the high informativity observed for PC1 allowed for single-component PCR (Blanco Romía & Alcalà Bernàrdez, 2009 ▸) as a quantitative model. Instead, the PLS method (Wold *et al.*, 2001 ▸) was used on the **IN-SS** data. Mean centring was applied prior to both PCA and PLS calculations.

All chemometric calculations were performed with Python (Version 3.11.7) scripts (Anaconda Software Distribution, Version. 2-2.4.0, https://anaconda.com) with PCA and PLS calculation based on the *scikit-learn* package (Pedregosa *et al.*, 2011 ▸).

Component selection for the PLS models was performed by evaluating the root-mean-square error in cross validation (RMSECV) (Wiklund *et al.*, 2007 ▸) of the increasing *n*-factor models:
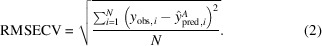
Leave one out cross validation was performed. The factor number (*A*) associated with the minimum error was considered, after evaluation of the loadings profile to verify the significance of all selected factors. The calibration error of the final model was then evaluated according to 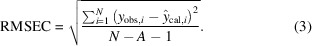
Here, *N* is the number of calibration samples, *y*_obs,*i*_ is the observed response for the *i*th sample, and 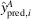 and 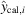 are the associated prediction in cross validation with *A* components and in calibration, respectively.

Lastly, the prediction ability of the models was evaluated with test sets composed of samples with FA fractions of 0.3 and 0.5 for the **NA-SS** crystals and 0.3, 0.5 and 0.7 for the **IN-SS** system. Each composition was tested in duplicate with independent synthesis. The prediction error for an observation *o* was calculated according to 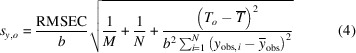
for the **NA-SS** model, since the proposed PCR model with only one component was considered equivalent to a univariate regression. In this instance RMSEC is the calibration error of the model [equation (3)], *b* represents the slope of the univariate regression, *M* is the number of measurements performed on the observation, *T*_*o*_ is the associated PC1 score and 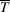 is the average PC1 score of the calibration set. A comparison with the multivariate approach confirms a comparable estimation of the error.

We stress that the evaluation of prediction uncertainty for multivariate models is still an open topic and no standard formula has been defined; both Zhang & Garcia-Munoz (2009 ▸) and Giussani *et al.* (2024 ▸) cite the reduced formula proposed by Faber & Kowalski (1996 ▸) [equation (5)] as a good solution for the estimation of multivariate variability, under the assumption that the signal measurement errors and the measurement error in the reference concentration can be neglected (Giussani *et al.*, 2024 ▸). This approach was applied, for example, by Skou *et al.* (2017 ▸). The assumptions required for the validity of the formula were considered satisfactory, as the current work is focused on a feasibility study; therefore prediction errors for the PLS models were calculated according to 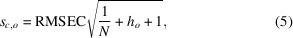
where *h_o_* = 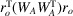 is the leverage for an individual observation *o*, *r*_*o*_ is the respective mean centred predictor vector, *W_A_* is the PLS weight matrix of the *A* components and *N* is the number of calibration samples. The term 1/*N* is due to mean centring.

## 3. Results and discussion

The aim of the present work is to assess the feasibility of a chemometric approach (i) to quantify the composition of a solid solution and (ii) to investigate the issues related to the use of PXRD data for this type of application. Solid solutions selected as model systems were obtained by reaction of IN with both FA and SA, which independently form isostructural co-crystals with isonicotinamide in the 2:1 stoichiometric ratio; reaction of IN with FA and SA was reported by Oliveira *et al.* (2008 ▸) to yield the solid solution IN_2_·FA_*x*_SA_1−*x*_ (**IN-SS**) with complete miscibility of FA and SA over the whole composition range. The change from the single –CH_2_—CH_2_– to the double –CH=CH– bond in the di­carb­oxy­lic acids does not significantly alter the steric requirements of the molecule, thus allowing for continuous replacement of one acid by the other with a small change in the crystal structure. No conformational change is observed between the two limit structures, despite the higher degrees of freedom of the single bond, ensuring isostructurality and facilitating substitution and full miscibility. The **IN-SS** system will be presented in the second section of this work, covering the internal standard and instrumental alignment combined with the PLS quantitative models.

**Figure 2:**
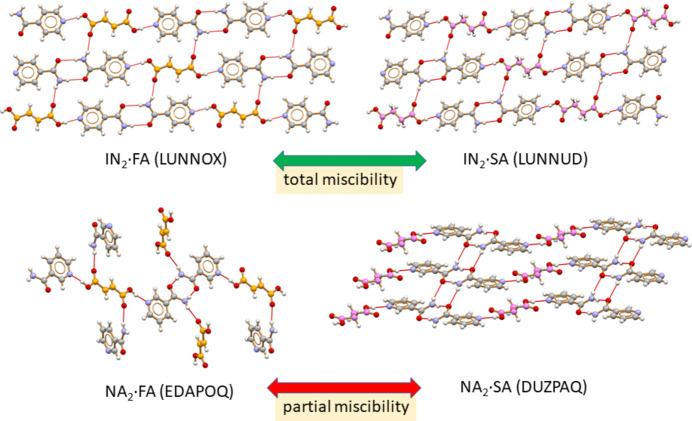
Relevant hydrogen-bonding interactions directing the packing arrangements in isomorphous/isostructural IN_2_·FA and IN_2_·SA (Aakeröy *et al.*, 2002 ▸) (top) and in non-isostructural NA_2_·FA (Orola & Veidis, 2009 ▸) and NA_2_·SA (Thompson *et al.*, 2010 ▸) (bottom) [carbon atoms in grey (IN and NA), orange (FA) and violet (SA)].

In order to prove the robustness of our analytical model applied to solid solutions, we also investigated the behaviour of nicotinamide, an isomer of isonicotinamide, which is known to form co-crystals with both SA [CSD (Cambridge Structural Database, https://www.ccdc.cam.ac.uk/) refcode DUZPAQ, 2:1 stoichiometric ratio; Thompson *et al.*, 2010 ▸] and FA (CSD refcodes EDAPOQ and NUKYAU for the 2:1 and 1:1 stoichiometric ratios, respectively; Orola & Veidis, 2009 ▸; Karki *et al.*, 2009 ▸). Solid solutions of formula NA_2_·FA_*x*_SA_1−*x*_ (**NA-SS**), containing NA and both co-formers SA and FA, were prepared, but in this case limited solubility was observed, and only solid solutions in the 0 < *x* < 0.6 range could be obtained. This might be expected as the two extremes, *i.e.* NA_2_·FA and NA_2_·SA, are not isostructural, as is the case with the iso­morphous isonicotinamide co-crystals IN_2_·FA and IN_2_·SA (Fig. 2 ▸).

### 3.1. Nicotinamide and succinic acid/fumaric acid solid solution NA_2_·FA_*x*_SA_1−*x*_ (NA-SS)

#### 3.1.1. Solid solution characterization

**Figure 3:**
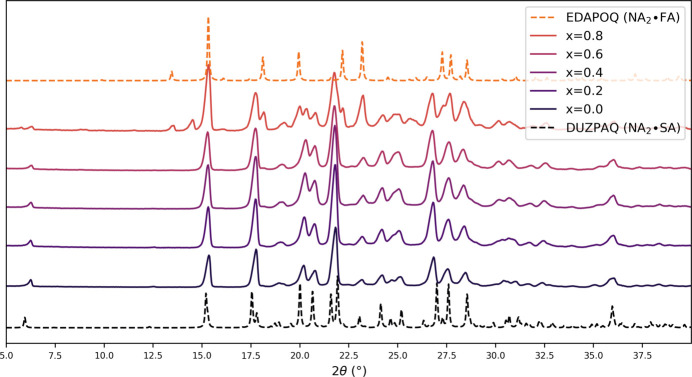
Experimental PXRD patterns (solid lines) for the solid solutions NA_2_·FA_*x*_SA_1−*x*_ (**NA-SS**) as obtained by ball-milling synthesis. For comparison, calculated patterns from single-crystal data are also shown (dashed lines) for the pure co-crystals NA_2_·SA (bottom, data collected at 120 K, CSD refcode DUZPAQ; the difference between the acquisition temperature and the room-temperature experimental data is responsible for the shift observable at higher 2θ) and NA_2_·FA (top, CSD refcode EDAPOQ).

**Figure 4:**
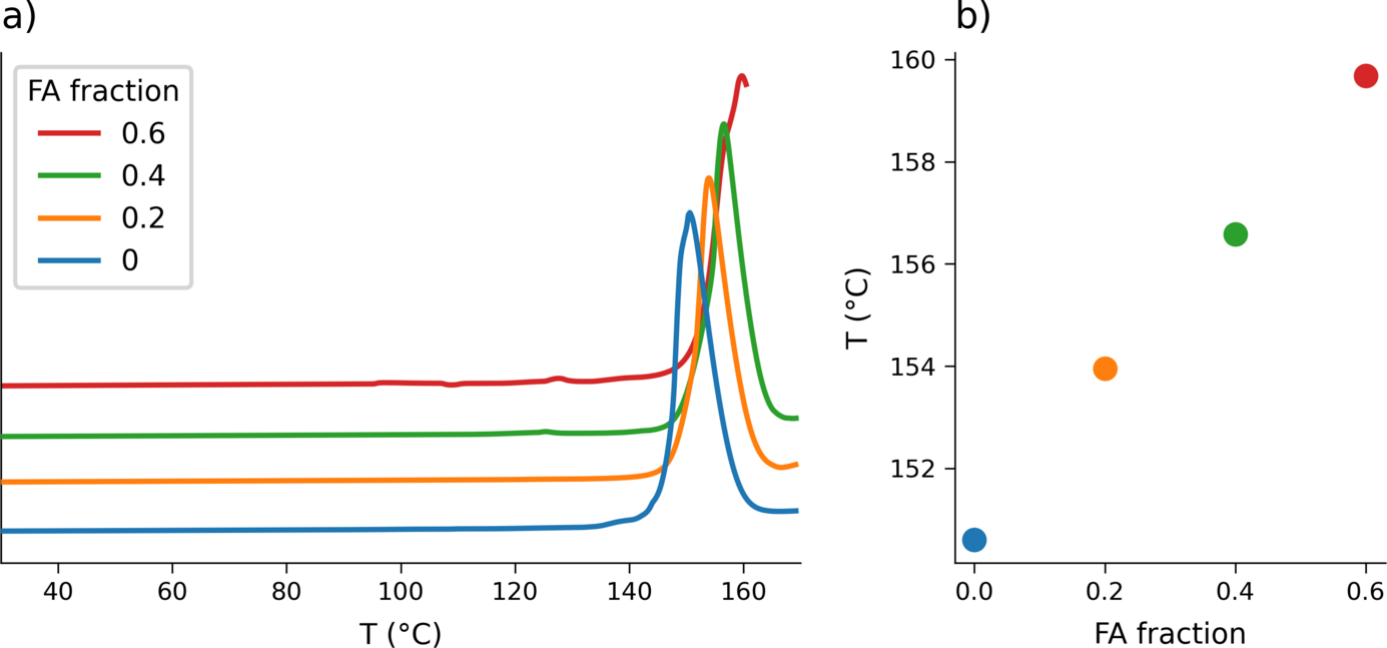
(*a*) DSC profiles for **NA-SS** (*x* = 0, 0.2, 0.4 and 0.6). (*b*) Scatter plot showing the linear behaviour of the melting peak values, taken from the DSC traces on the left, versus the FA molar fraction *x*.

Powders of **NA-SS** were obtained via mechanochemistry as described in Section 2.2. The XRD patterns show a constant profile (Fig. 3 ▸) with no visible traces of the starting materials, matching the pure NA_2_·SA pattern up to an FA content of 0.6, suggesting the formation of a solid solution. As a further verification, DSC analysis was performed, showing a single endothermic event and a linear progression of the melting point (Fig. 4 ▸) consistent with the formation of a single phase with modulated physical properties, as with the case of solid solutions. The incomplete curve observed for the 0.6 FA sample was due to the degradation of the material, which forced the interruption of the measurement. This behaviour suggests a higher instability of the structure at this composition, consistent with the limit of miscibility that was observed for the system by PXRD.

**Figure 5:**
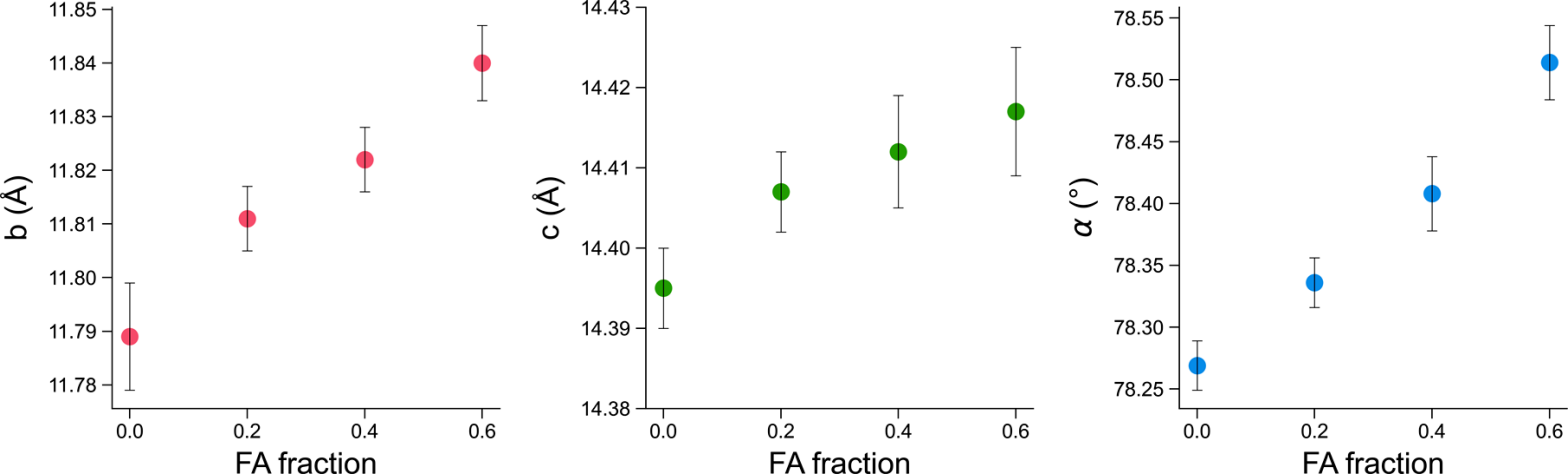
Plots of selected unit-cell parameters (*b* and *c* lengths, and the α angle) resulting from Pawley analyses of PXRD profiles collected for **NA-SS** at different values of the FA molar fraction *x*. Error bars correspond to errors returned by the fit.

The effect on the cell parameter values was assessed via Pawley refinement. Fig. 5 ▸ shows the gradual variation in cell parameters accompanying the increasing fraction of FA, as is expected for solid solutions. The crystallographic *b* axis and α angle are those most affected by the substitution, with the *b* length expanding up to 0.05 Å for *x* = 0.6 (FA fraction) and the α angle increasing by 0.24° (additional data in Section S6). The expansion of the unit cell is at the basis of the possibility of tracing the solid solution composition through the PXRD profile: as a consequence of variations in cell parameters, shifts in the peak 2θ values are also observed, which can be used as signals for the quantitative model.

#### 3.1.2. Alignment method selection

**Figure 6:**
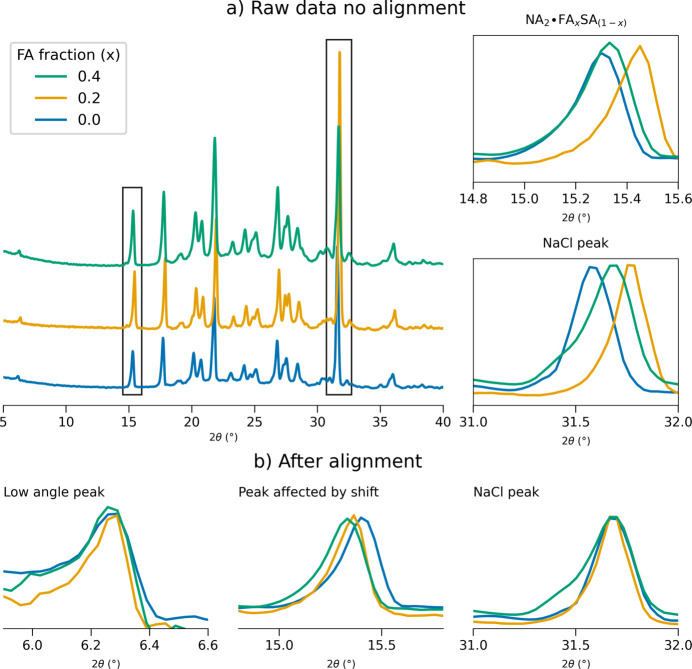
Experimental patterns of three **NA-SS** samples (*x* = 0, 0.2 and 0.4) with the addition of the internal standard NaCl. (*a*) Before manual alignment. (Left) Full patterns and (right) detail of the two peaks highlighted by the black rectangle, belonging to (top) the solid solution profile and (bottom) the NaCl profile. (*b*) After manual alignment. From left to right: solid solution peaks at 6.3° and 15.2°, and peak from internal standard NaCl.

Cell parameters are not the only factors influencing the peak positions; some experimental factors, such as sample displacement and sample transparency (Parrish & Langford, 2006 ▸), can also affect peak position, potentially interfering with the signal under examination. While Pawley and Rietveld refinements evaluate peak positions via profile fitting and are able to compensate for experimental factors with specific coefficients, a chemometric calculation, applied directly to the raw experimental data, requires an alignment strategy to compensate for the experimental variability. One possibility for alignment is of course data acquisition in transmission, while in Bragg–Brentano geometry (*i.e.* the commonly available reflection geometry for standard laboratory instrumentation) the primary source of the 2θ shift, expressed by equation (1), is from the sample surface not being tangential to the focusing circle. Evidence of this can be found in the profiles shown in Fig. 6 ▸, where acquisitions from three solid solution compositions are compared for the **NA-SS** system mixed with NaCl.

**Figure 7:**
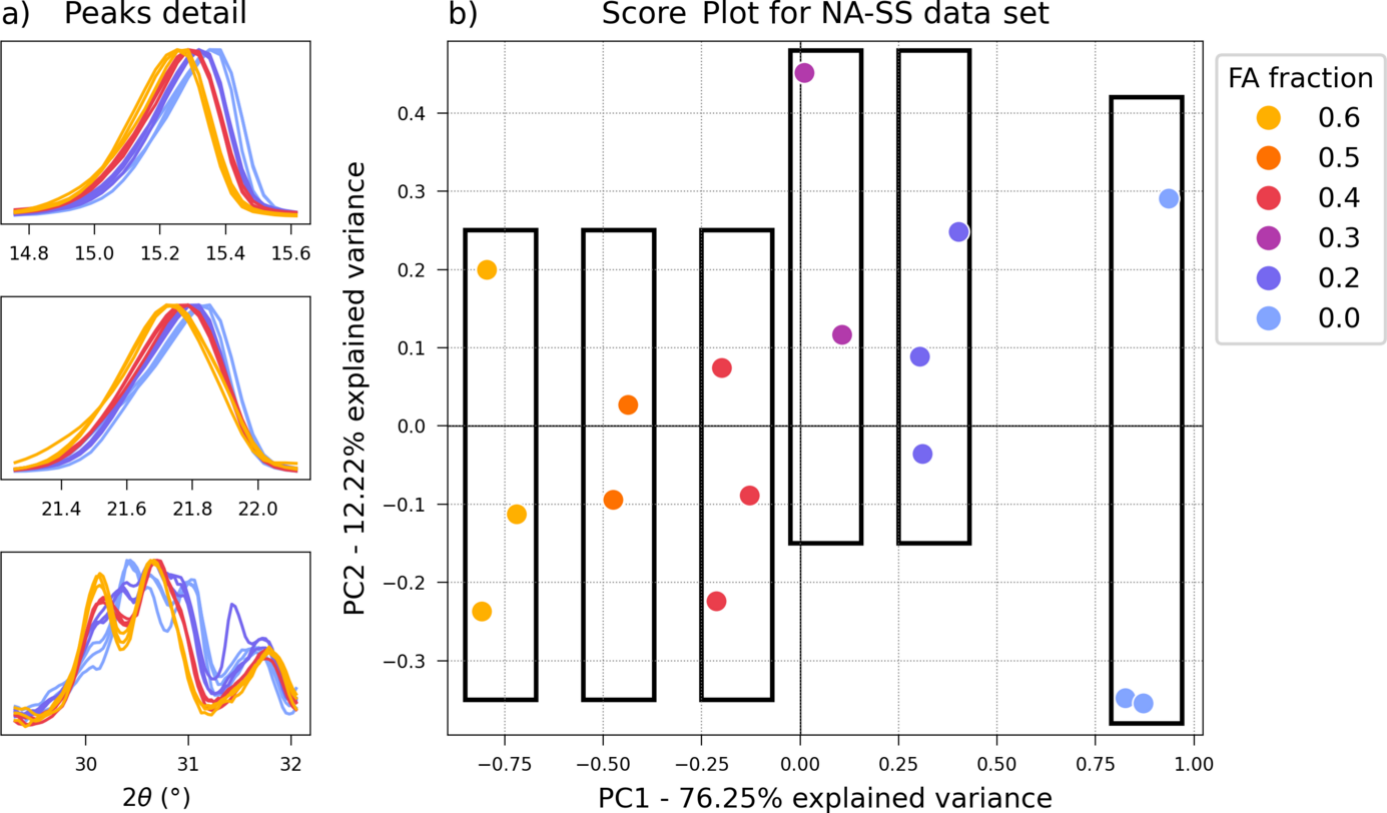
(*a*) Detail of experimental patterns of the solid solution **NA-SS** (*x* = 0, 0.2, 0.4 and 0.6), showing peaks affected by a shift (top and centre) or progressive change (bottom) according to the FA:SA ratio. (*b*) Score plot (PC1 versus PC2) of the full **NA-SS** data set after manual alignment.

While we expect continuous peak shifts based on solid solution compositions, the 2θ values of the NaCl peaks are expected to remain constant if the sample is correctly aligned. Therefore, the most intense peak of NaCl [2θ = 31.7°, Fig. 6 ▸(*a*)] can be used as an internal reference to verify the presence of sample displacement for all measurements. Fig. 6 ▸(*a*) shows a comparison of untreated acquisitions, evidencing an incorrect progression of the solid solution peaks (top) and an evident divergence of the NaCl peak for all three samples (bottom). If the NaCl peak of the 0 and 0.2 curves (blue and orange lines respectively) is properly shifted to its ideal value, as shown in Fig. 6 ▸(*b*), the expected progression of the solid solution appears, as shown in the 2θ peak at 15.3° in the middle of the figure. Note that the Δ2θ correction for each aligned pattern was not larger than 0.1° which, from equation (1), corresponds to a deviation of about 0.2 mm of the sample surface from the ideal position, *i.e.* tangential to the focusing circle. Even with extremely careful sample preparation it is not an easy task to maintain such control of the sample holder filling, especially in this specific case, as the limited expansion of the cell due to formation of the solid solution causes shifts of the order of 10^−2^° (see below), thus sensitive to a displacement of the sample surface of 10^−2^ mm. Therefore, PXRD measurements in reflection require an alignment strategy for the purpose of quantitative applications. For the nicotinamide system the alignment with NaCl revealed the presence of a characteristic peak not affected by the solid solution progression, corresponding to the first reflection at 2θ = 6.3°; as shown in Fig. 6 ▸(*b*) (left), all fractions appear aligned at this value. The low-angle peak was thus considered as the reference peak for the alignment and no internal standard was further added to the samples. The alignment procedure of the final data set was verified by observing the overlap of the reference peaks, which was consistent for all samples (Fig. S1). In Fig. 7 ▸(*a*) a selection of peaks are reported (with more in Fig. S1), showing the expected progression of the 2θ shift with good overlap between replicates, which indicates reproducibility of the synthesis and method of analysis. Statistical analysis carried out on the profiles returned a mean standard deviation of the 2θ peak positions of 0.006° (evaluated for the first 13 peaks of the profile, Table S1). Moreover, considering the peak mean position for each fraction, it was possible to estimate the magnitude of the shift, resulting in a 2θ mean value of 0.03° per step over 13 peaks (full data available in the supporting information, Section S1).

#### 3.1.3. Multivariate analysis (PCA and PCR)

**Figure 8:**
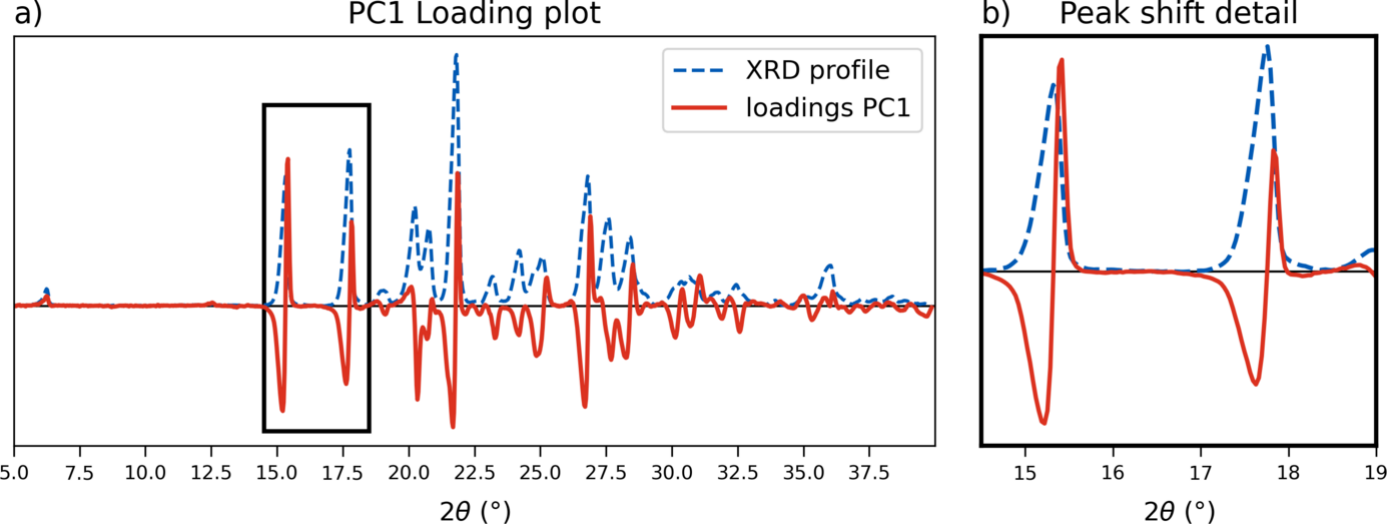
PC1 loadings plot (red solid line) from PCA analysis of the full **NA-SS** data set compared with the original PXRD profiles (blue dashed line). (*a*) Full profile and (*b*) detail of the 14.5–19° 2θ range, highlighted by the black rectangle in the full profile.

Once a data set had been prepared as described in Section 2.5, an exploratory analysis of both training and test samples was first carried out with PCA to test the ability of a multivariate analysis to observe the progression of the solid solutions. In Fig. 7 ▸(*b*) the score plot of the first two components is reported, where an evident progression of the samples depending on their composition is observed. The desired separation occurs along the PC1 direction, with samples aligning at specific PC1 values according to their FA content: higher values of fumaric fraction correspond to negative PC1 scores. In contrast, PC2, while contributing 12.22% of the explained variance, does not appear significant for the desired characterization. Confirmation of this can be found on the loadings plot, where the contribution of the 2θ angles to the separation can be identified. In Fig. 8 ▸ the PC1 loading profile is compared with the original diffractogram, and a distinctive trend is visible where characteristic peaks in the PXRD pattern correspond to a split peak in the loadings: the left side contributes to a negative loading and the right side to a positive loading. This behaviour matches closely the experimental solid solution shift, where at higher FA content the peaks are found at lower 2θ angles (shifted to the left), while with lower FA content the PXRD profile is shifted to the right. As a result, PC1 scores decrease accordingly with the increase in FA. In contrast, the PC2 loadings profile (Fig. S8) shows a non-characteristic split and matches the PXRD profile, therefore accounting for experimental variability, assigning negative values to a general increase in intensity.

**Figure 9:**
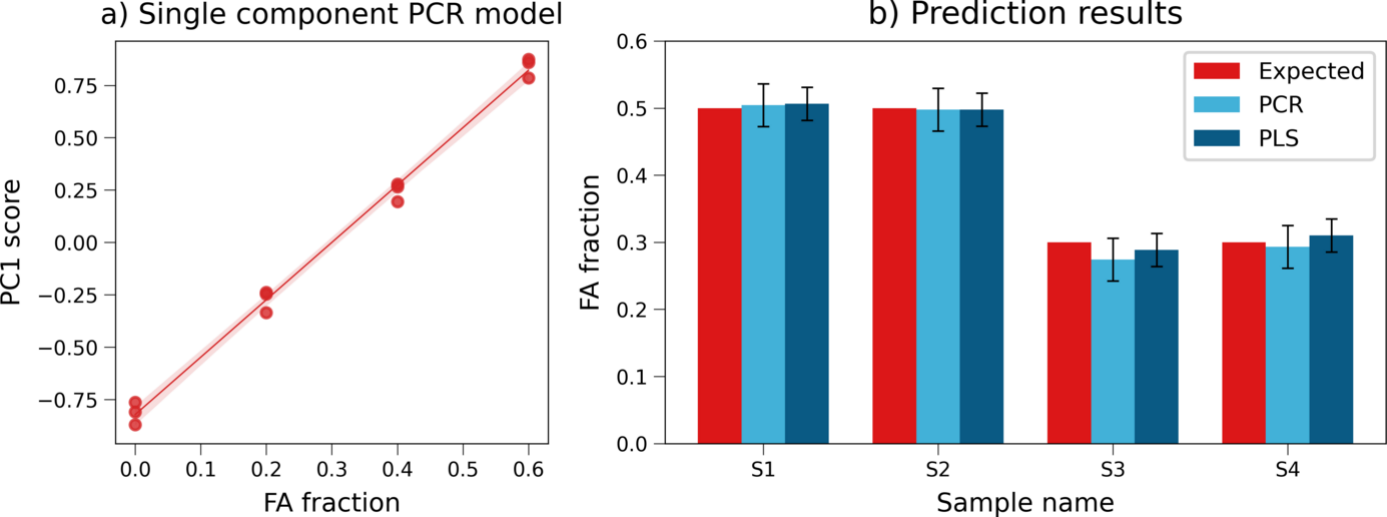
(*a*) One-component PCR regression line (PC1 versus FA fraction) for the **NA-SS** data set. (*b*) Comparison of predicted FA fraction obtained with PCR regression in panel (*a*) (light blue) and a full multivariate PLS model with three components (dark blue) relative to control samples with expected FA fractions of 0.5 (S1 and S2) and 0.3 (S3, S4). Expected composition is plotted as a reference in red.

The high informativity of PC1 (i) allows for a rapid identification of the solid solution behaviour, since a wider peak split of the loadings is related to a higher shift in the XRD profile (more details in Section S1), and (ii) can provide an easy approach for a quantitative model where the PC1 score is correlated with the solid solution composition. The steps of the PCR model, built using the training data set (three replicates of *x* = 0, 0.2, 0.4 and 0.6), were the following. First the PCA calibration model was built (Fig. S9), which maintained analogous features to those observed for the full data set; the resulting PC1 scores were correlated to the FA fraction in a univariate linear regression. The obtained regression line [Fig. 9 ▸(*a*)] has intercept 2.74 ± 0.06, slope −0.82 ± 0.02 (significance 0.05), *R*^2^ 0.995 and RMSE 0.05. The model was then tested for its prediction ability using control samples with FA contents of 0.3 and 0.5, each from two independent syntheses. A comparison of the obtained values (0.27 ± 0.03, 0.29 ± 0.03, 0.50 ± 0.03 and 0.50 ± 0.03) with the expected values on the bar plot in Fig. 9 ▸(*b*) shows that the model is able to correctly identify the solid solution composition. The dark-blue series in Fig. 9 ▸(*b*) instead shows the results obtained with a three-component PLS model. Both the prediction and standard deviation of the PCR model are well matched, supporting the validity of the simpler approach and the univariate method for error estimation.

#### 3.1.4. Automatic alignment (Python)

To explore the possibility of a fully automated model, background correction and alignment were further integrated in the Python script (Section 2.5.1). Comparable results to those of the manual procedure were obtained once the correct alignment strategy was identified, which consisted of two main steps: (i) detection of the reference peak, requiring a constant mapping of the profile for correct location of the alignment peak and its 2θ position within all samples, and (ii) definition of the alignment method, sensitive to the peak shape.

For the **NA-SS** data set the reference peak is the first in the profile and this simplified the initial step, whereas for the **IN-SS** data set, where the reference peak is further along the pattern, an optimization of the prominence and width parameters of the find_peak function can be necessary to match the alignment peak correctly in all samples.

**Figure 10:**
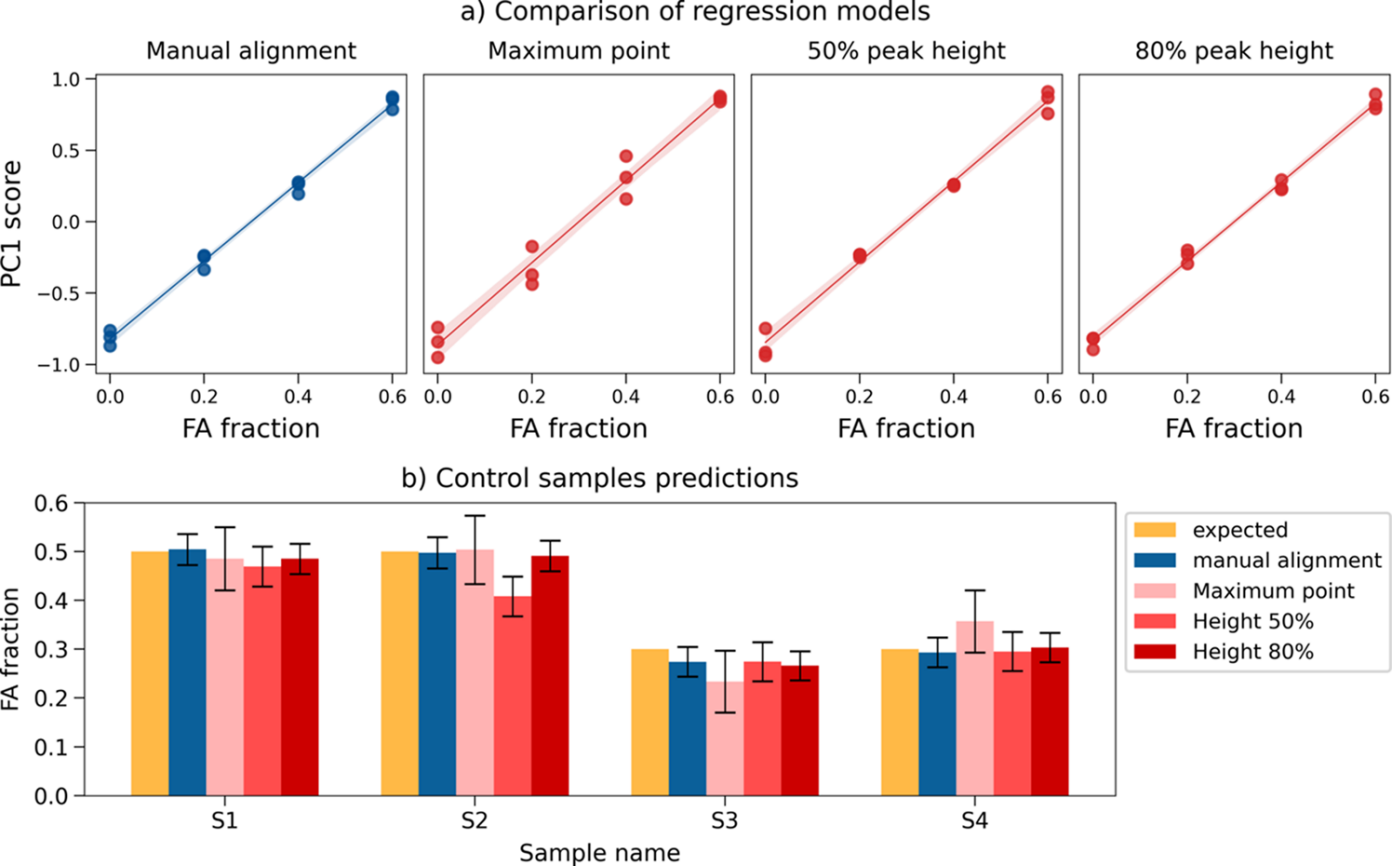
(*a*) Comparison of regression models resulting from the **NA-SS** calibration data set after different alignment strategies. Regression relative to the manual procedure (blue) on the left-hand side as reference; regressions resulting from automated alignment (red) follow, with peak position calculated as (from left to right) the point of maximum intensity, the middle point of width at half-height and the middle point of width at 80% of peak height. (*b*) Comparison of the predicted FA fraction returned by the regression models in Fig. 8(*a*) for the four control samples tested. The first series (yellow) shows the expected concentrations.

The second step was more critical. For the alignment correction the 2θ difference between the position of the reference peak in the first sample and the subsequent patterns is calculated so that the second profile can be shifted accordingly; for this reason, determination of the peak position is essential to the procedure. The find_peaks function returns by default the position of the point with maximum intensity which, due to experimental noise, often does not correspond to the centre of the peak, causing an incorrect alignment (examples are shown in Section S2). To detect the centre of the peak correctly, the middle point of the peak width was considered; this, however, can be affected by an asymmetry in the peak shape (Fig. S5). The peak width was then evaluated at different heights: a value of 80% was found to allow an alignment similar to that obtained via the manual procedure. The impact of the peak detection can be seen in Fig. 10 ▸, where the models [Fig. 10 ▸(*a*)] and prediction results [Fig. 10 ▸(*b*)] are compared for the manual (blue) and automated alignment procedures (red). Data for the automated method correspond to peak detection at the maximum point and at 50% and 80% of the height. The first two methods show a higher variability in both the model and the control sample predictions, while the 80% height gave results comparable to the manual results.

### 3.2. Isonicotinamide–succinic acid/fumaric acid (IN-SS)

**Figure 11:**
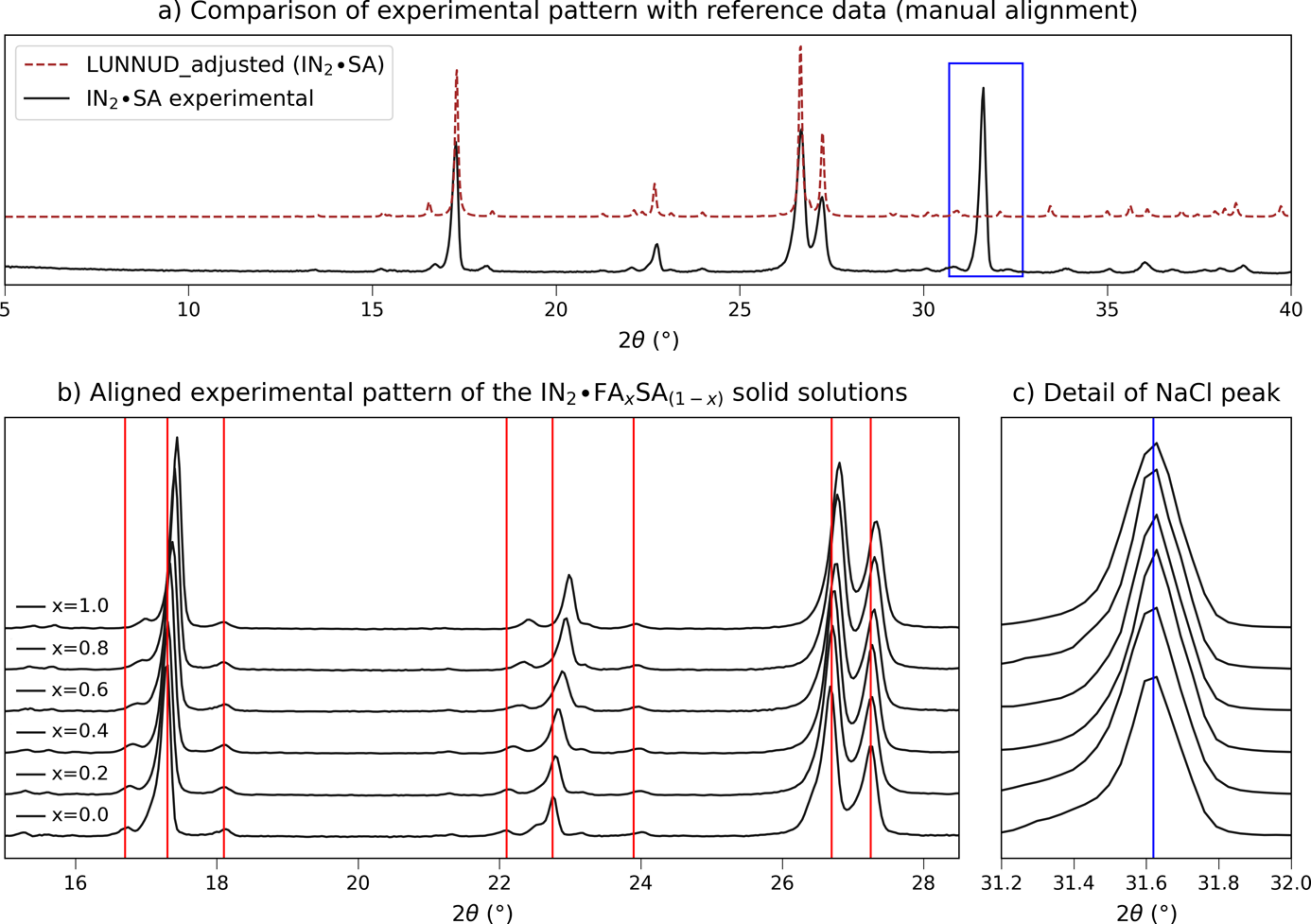
Experimental PXRD data for the **IN-SS** data set after manual alignment. (*a*) Comparison of the experimental profile (solid line) with the calculated pattern from single-crystal data (dashed line). The pattern calculated on the basis of single-crystal data (CSD refcode LUNNUD, data collected at 173 K), shifted as a result of cell thermal contraction with respect to ambient conditions (see the supporting information), was optimized with the *AutoFidel* algorithm (*Mercury* program from the Cambridge Crystallographic Data Centre; https://www.ccdc.cam.ac.uk/solutions/software/mercury/; Macrae *et al.*, 2020 ▸). The NaCl peak, used as internal standard, is highlighted with the blue rectangle. (*b*) Details of the 15–28° 2θ range of the experimental patterns of solid solutions with increasing FA fraction (*x*). The vertical red lines help trace the shift affecting the peak positions. (*c*) Detail of the NaCl peak.

Given the satisfactory results obtained with the **NA-SS** system, the quantitative approach was further tested on the isonicotinamide solid solution IN_2_·FA_*x*_SA_1−*x*_ (**IN-SS**) which presents a full range of miscibility. Due to the characteristics of the diffraction pattern, in this case different alignment strategies were adopted. The PXRD profile obtained for the compound is reported in Fig. 11 ▸(*a*); it matches the calculated pattern (CSD refcode LUNNUD; Aakeröy *et al.*, 2002 ▸) except for the peak from NaCl (outlined with a blue rectangle), which was added to the sample for the preliminary alignment. The profile is characterized by fewer high-intensity peaks and no significant reflections are present before 15° 2θ. This condition reduces the possibility of finding peaks not affected by the cell expansion and suitable as reference for the alignment. In Fig. 11 ▸(*b*) the progression of the aligned XRD profiles at different compositions is reported, and a shift is noticeable for all main peaks, as highlighted by the vertical lines. Only the reflection at 18.1° 2θ appears less influenced by the progression, but the characterization presented in the original paper on the calculated patterns (Oliveira *et al.*, 2008 ▸) also shows a contribution of the expansion on this peak, so it was excluded as a reference. In Fig. 11 ▸(*c*) the detail of the NaCl peak is reported as evidence of the correct alignment. Since no element of the solid solution profile was unaffected by the transformation, NaCl was adopted as an internal standard and maintained for the development of the quantitative model.

The **IN-SS** samples were also analysed with the Empyrean diffractometer equipped with the *XYZ* sample stage (Section 2.4); this setup was able to correct the displacement of the sample holder by finely adjusting, with a laser sensor, the *Z* coordinate of the sample stage. These measurements were used to build a second data set that, thanks to the instrumental alignment, did not require any additional 2θ correction. The samples used in this case were the same ones containing the internal standard so as to give a rapid verification of the correctness of the alignment. In Fig. S7 the full data set and the NaCl peak detail are reported.

#### 3.2.1. Quantitative model (PLS)

**Figure 12:**
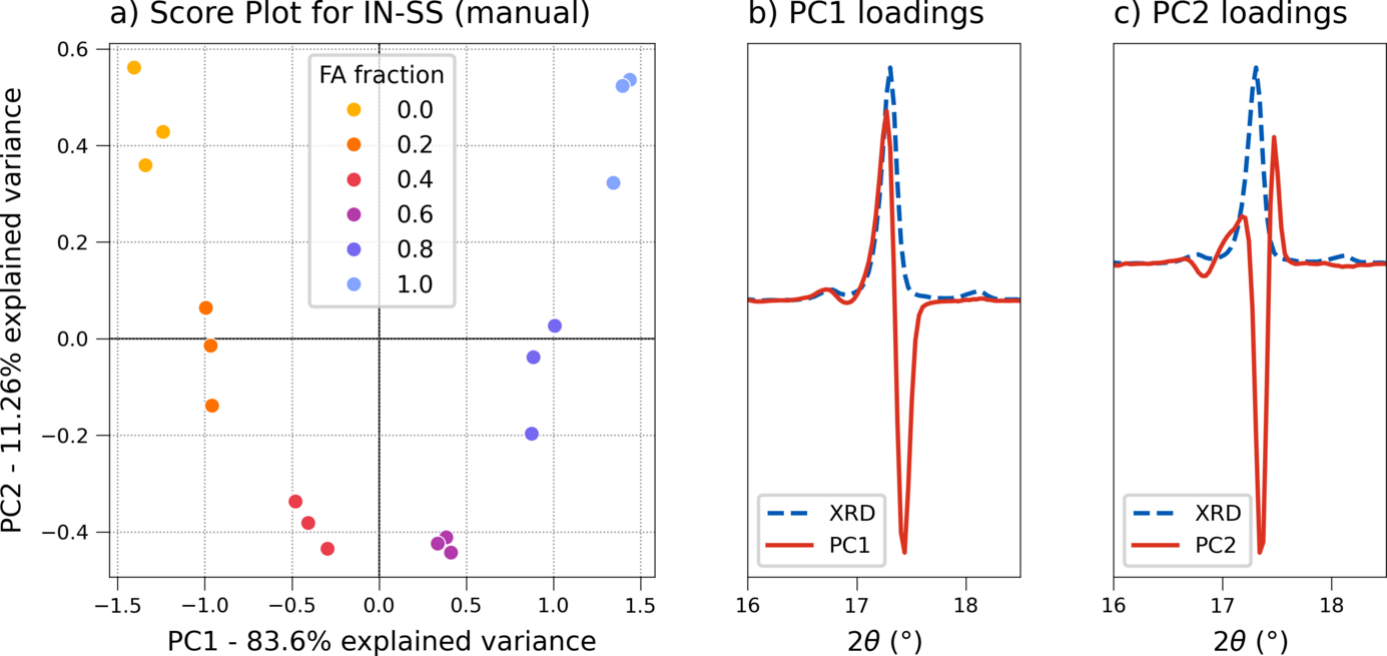
(*a*) Score plot (PC1 versus PC2) of the **IN-SS** calibration set (manual alignment). Details of the relative loadings plots for PC1 and PC2 are reported in panels (*b*) and (*c*), respectively (red solid lines), and compared with the experimental XRD profile (blue dashed lines) (see Fig. S10 for the full loadings profiles).

PCA exploratory analysis was first carried out on the two **IN-SS** data sets [Fig. 12 ▸(*a*) and Fig. S11]. The solid solution progression was visible in both sets, pointing to a good alignment, but the PC1 component was not solely responsible for the separation; the PC2 direction also appeared relevant. The samples are aligned along the *x* axis but form distinct clusters along the *y* direction, with the intermediate fractions found at negative PC2 values and the pure co-crystals placed at high positive scores. Loadings plots (full data in Fig. S10) confirm the observations: the PC1 profile shows the characteristic peak split feature [Fig. 12 ▸(*b*)] observed with the previous system, but the PC2 component also presents a similar trend [Fig. 12 ▸(*c*)], in which the central contribution of the peak is assigned to negative loadings and the edges to a positive value. Therefore both components appear related to the 2θ shift, suggesting a more complex evolution of the XRD profile that is not easily represented by a single component; the higher complexity could be due to the full miscibility of the system, causing a higher 2θ shift across the sequence and so a higher total variability. Since more than one principal component was needed to describe the system, the PCR approach was not considered for the quantitative model, but a PLS regression was chosen instead. The PLS algorithm searches for component directions while maximizing the correlation with the response, making for a more robust quantitative model.

**Figure 13:**
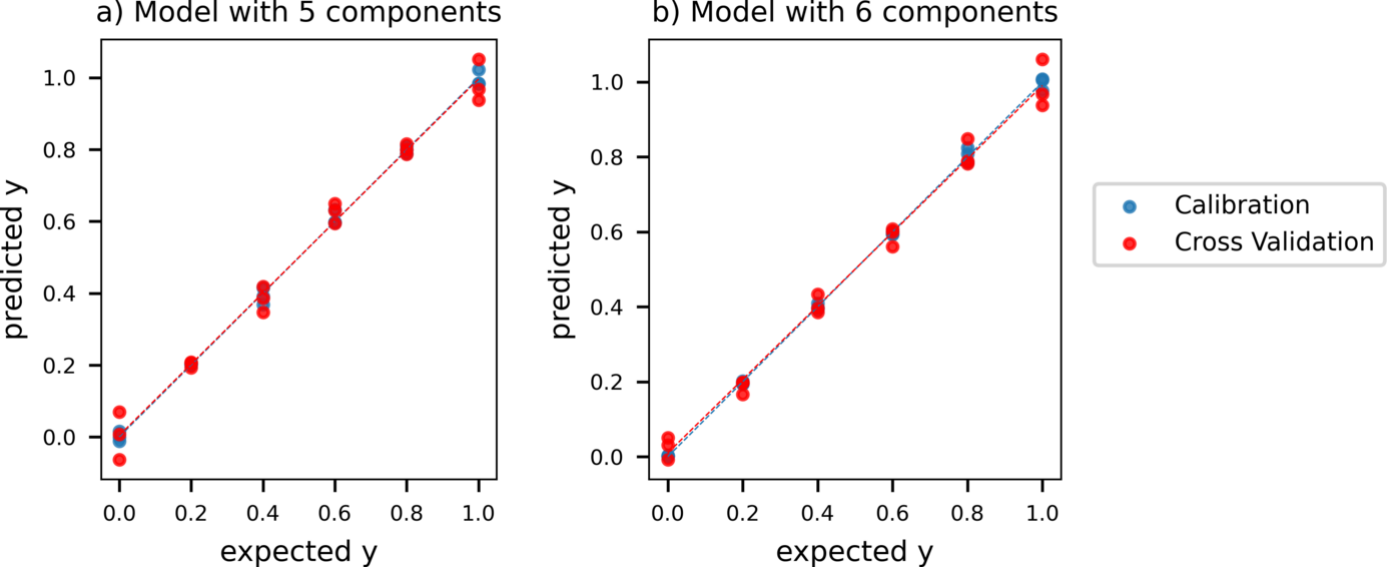
Parity plots of the final **IN-SS** PLS model from (*a*) the manual alignment and (*b*) the instrumental alignment data sets.

The PLS model was then evaluated (Section 2.5.3), presenting very similar features for both data sets: despite the main contribution to the explained variance being found in the first factor (97%), the lowering of the error in cross validation (CV) appears significant up to the fifth and sixth factors for the manual and Empyrean data sets, respectively. The loadings plots show a variation of the split peak trend, with low noise contribution, for all selected components, pointing to the relevance of all factors (Fig. S12). Therefore, a five-factor model was selected for the manual data set [Fig. 13 ▸(*a*)] having an RMSECV of 0.0395 and a good correlation: *R*^2^ values were 0.998 and 0.986 for calibration and CV, respectively. The parity plot in Fig. 13 ▸(*b*) corresponds to the six-component model of the instrumental alignment data set, associated with an RMSECV of 0.0356, with *R*^2^ of 0.999 in calibration and 0.991 in CV.

**Figure 14:**
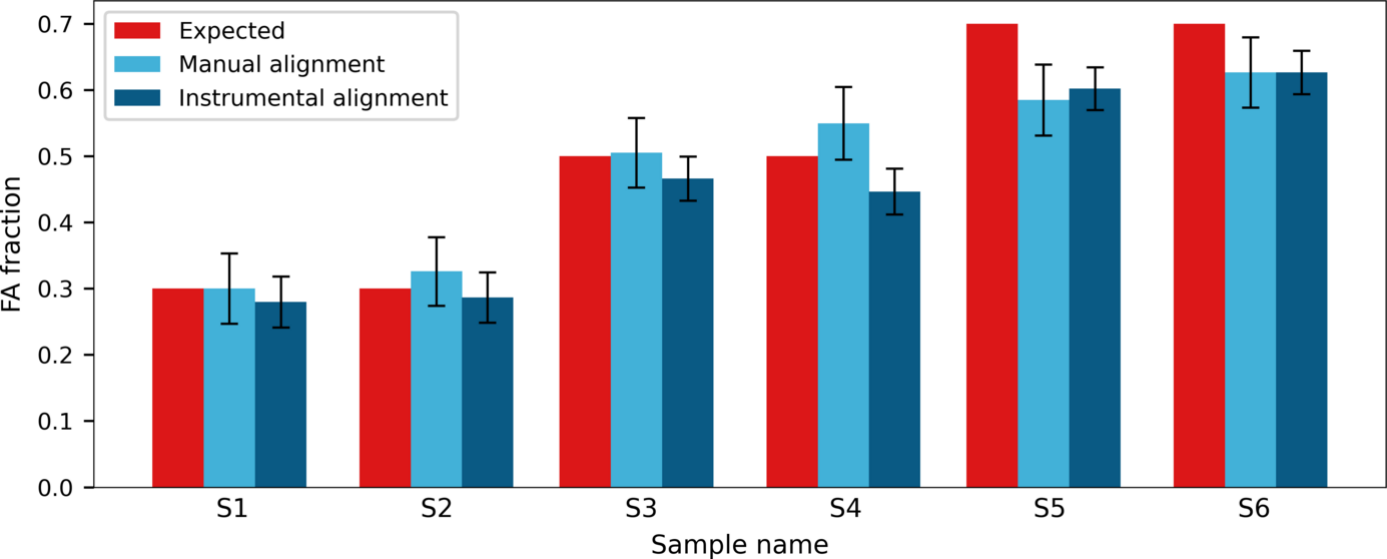
Comparison of predictions from the manual (light blue) and instrumental (dark blue) alignment models for six control samples of the **IN-SS** system. The expected FA content (red) was 0.3 for samples S1 and S2, 0.5 for samples S3 and S4, and 0.7 for samples S5 and S6.

**Figure 15:**
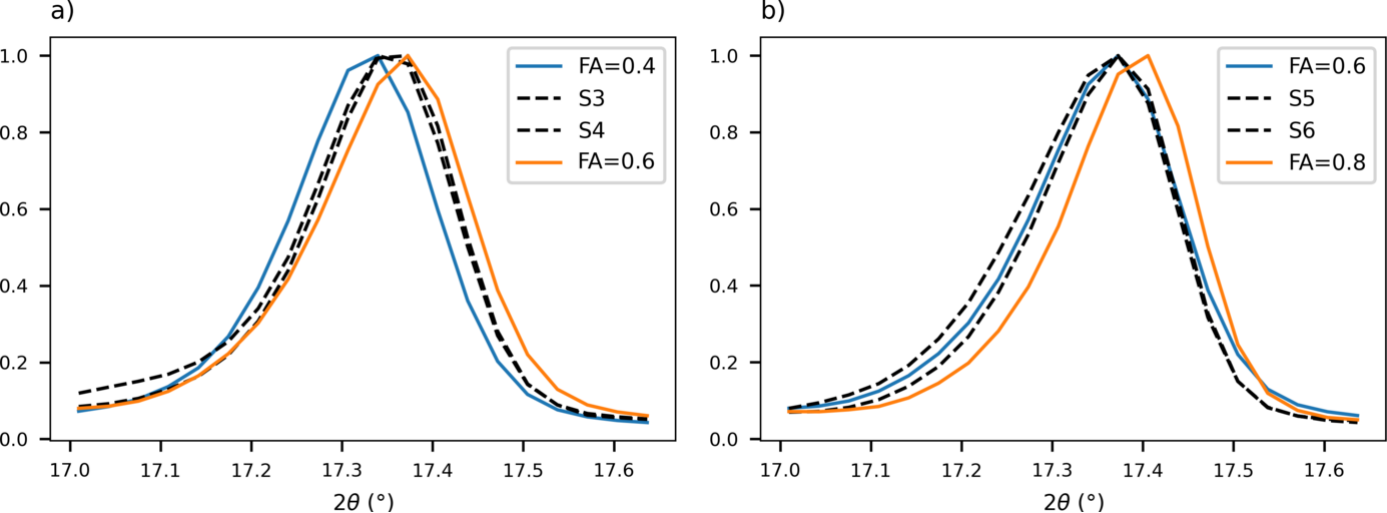
The 17.3° 2θ peak from experimental PXRD patterns of control samples (dashed lines) and calibration data (solid lines) for the **IN-SS** manual data set. (*a*) Control samples with 0.5 FA (S3, S4) and calibration samples with 0.4 and 0.6 FA. (*b*) Control samples with 0.7 FA (S5, S6) and calibration samples with 0.6 and 0.8 FA.

Lastly, model validation was carried out on control samples with 0.3, 0.5 and 0.7 FA fractions. Fig. 14 ▸ reports comparisons between predictions obtained with the two PLS models, showing good agreement between the two alignment techniques. The manual alignment is related to a slightly higher variability in the prediction with a mean prediction error of 0.05, versus 0.03 for the instrumental alignment; since the main contribution to the error is given by the residuals in the calibration, it is reasonable to observe a higher inaccuracy from the manual approach. However, the predicted values appear comparable for all samples, agreeing with the expected concentration for both 0.3 and 0.5 fractions (S1, S2, S3, S4), while the 0.7 samples (S5, S6) are underestimated by both models. On closer inspection of the aligned diffractograms [Fig. 15 ▸(*b*)], both 0.7 samples appear aligned with the 0.6 fraction, instead of being positioned in between the 0.6 and 0.8 fractions as expected, while, for example, the 0.5 fraction is correctly found between the 0.4 and 0.6 samples [Fig. 15 ▸(*a*)]. This observation suggests that the underestimation of the predictions is due to the sample and not to any limited prediction capability of the models; from the model perspective, the result of the prediction is not incorrect, since the profile evaluated is indeed indistinguishable from the 0.6 fraction sample. Since this characteristic is observed for two independent syntheses, it can be hypothesized that the behaviour is not due to experimental error, either in the synthesis or in the acquisitions; data from the original characterization of the system (Oliveira *et al.*, 2008 ▸) also showed a variability in the progression between fractions 0.5 and 0.7, suggesting that, in that compositional range, unit-cell parameter modifications and related peak shifts could be less evident. This condition could influence the model accuracy for the specific system but, for a more general evaluation of the approach, the discrepancy in the prediction should not be regarded as a flaw in the methodology: as long as progression in the profile is present, it is possible to predict the sample composition correctly.

## 4. Conclusions

In this work a multivariate analysis approach was tested to predict solid solution composition using PXRD data. We have shown how PCA analysis of full PXRD profiles is able to detect the characteristic profile shift induced by cell expansion, revealing a sensitivity of the method to the subtle modifications caused by changes in solid solution composition, and have presented PCA as a tool for the easy identification of distinctive features of the powder pattern. Moreover, it was possible to calibrate the profile shift with multivariate linear regression methods, like PCR and PLS, showing the feasibility of developing quantitative models able to predict the composition of unknown solid solution samples from XRD profiles.

To prove the robustness of the approach, the methodology was tested on two organic solid solution systems obtained from the substitution of succinic acid with fumaric acid in the co-crystals they form with isonicotinamide (IN_2_·FA_*x*_SA_1−*x*_, **IN-SS**) or nicotinamide (NA_2_·FA_*x*_SA_1−*x*_, **NA-SS**) as co-former. The **NA-SS** system was here reported for the first time and characterized as a solid solution by PXRD, DSC and Pawley refinement. Good model statistics and prediction results were obtained in both cases, supporting the feasibility of the methodology proposed.

Diffraction patterns acquired in Bragg–Brentano geometry were used for all calculations, presenting an easy experimental procedure that only required caution in considering the experimental shift due to sample displacement. Different alignment strategies were proposed to tackle the problem, from the identification of unaffected reflections to the use of an internal standard or instrumental alignment. The best strategy was chosen on the basis of the specific profile characteristics, and in all cases allowed for the correct identification of the progression and the building of the model. The alignment procedure has been presented as both a manual pretreatment and an automatic analysis integrated into the chemometric calculation.

## Supplementary Material

Additional figures and table. DOI: 10.1107/S1600576725006910/ui5036sup1.pdf

## References

[bb64] Aakeröy, C. B., Beatty, A. M. & Helfrich, B. A. (2002). *J. Am. Chem. Soc.* **124**, 14425–14432. 10.1021/ja027845q 12452718

[bb1] Barr, G., Dong, W. & Gilmore, C. J. (2004). *J. Appl. Cryst.* **37**, 658–664.

[bb3] Blanco Romía, M. & Alcalà Bernàrdez, M. (2009). *Infrared spectroscopy for food quality analysis and control*, pp. 51–82. Elsevier.

[bb4] Braga, D. (2023). *Chem. Commun.* **59**, 14052–14062. 10.1039/d3cc04313d 37938038

[bb5] Braga, D., Casali, L. & Grepioni, F. (2022). *Int. J. Mol. Sci.* **23**, 9013. 10.3390/ijms23169013 PMC9408954 36012275

[bb6] Bruni, G. (1900). *Atti Real. Accad. Lincei* **9**, 232–241.

[bb7] Caliandro, R. & Belviso, D. B. (2014). *J. Appl. Cryst.* **47**, 1087–1096.

[bb8] Caliandro, R., Di Profio, G. & Nicolotti, O. (2013). *J. Pharm. Biomed. Anal.* **78–79**, 269–279. 10.1016/j.jpba.2013.01.042 23518290

[bb9] Chen, S., Xi, H., Henry, R. F., Marsden, I. & Zhang, G. G. Z. (2010). *CrystEngComm* **12**, 1485–1493.

[bb10] Cherin, P. (1973). *Applications of the newer techniques of analysis*, edited by I. L. Simmons & G. W. Ewing, pp. 103–122. Boston: Springer.

[bb11] Coelho, A. A. (2018). *J. Appl. Cryst.* **51**, 210–218.

[bb12] Cruz-Cabeza, A. J., Lestari, M. & Lusi, M. (2018). *Cryst. Growth Des.* **18**, 855–863.

[bb13] Dabros, M., Emery, P. R. & Thalladi, V. R. (2007). *Angew. Chem. Int. Ed.* **46**, 4132–4135. 10.1002/anie.200604830 17394267

[bb14] d’Agostino, S., Fornasari, L., Grepioni, F., Braga, D., Rossi, F., Chierotti, M. R. & Gobetto, R. (2018). *Chem. A Eur. J.* **24**, 15059–15066. 10.1002/chem.201803071 30011358

[bb15] Denton, A. R. & Ashcroft, N. W. (1991). *Phys. Rev. A* **43**, 3161–3164. 10.1103/physreva.43.3161 9905387

[bb16] Duggirala, N. K., Perry, M. L., Almarsson, Ö. & Zaworotko, M. J. (2016). *Chem. Commun.* **52**, 640–655. 10.1039/c5cc08216a 26565650

[bb17] Faber, K. & Kowalski, B. R. (1996). *Chemometr. Intell. Lab. Syst.* **34**, 283–292.

[bb18] Fonseca, J. C., Tenorio Clavijo, J. C., Alvarez, N., Ellena, J. & Ayala, A. P. (2018). *Cryst. Growth Des.* **18**, 3441–3448.

[bb19] Giussani, B., Gorla, G., Ezenarro, J., Riu, J. & Boqué, R. (2024). *TrAC Trends Anal. Chem.* **181**, 118051.

[bb20] Grepioni, F., Casali, L., Fiore, C., Mazzei, L., Sun, R., Shemchuk, O. & Braga, D. (2022). *Dalton Trans.* **51**, 7390–7400. 10.1039/d2dt00834c 35466980

[bb21] Guccione, P., Palin, L., Milanesio, M., Belviso, B. D. & Caliandro, R. (2018). *Phys. Chem. Chem. Phys.* **20**, 2175–2187. 10.1039/c7cp06326a 29104977

[bb22] Haque, A., Alenezi, K. M., Khan, M. S., Wong, W.-Y. & Raithby, P. R. (2023). *Chem. Soc. Rev.* **52**, 454–472. 10.1039/d2cs00262k 36594823

[bb23] Hasell, T., Chong, S. Y., Schmidtmann, M., Adams, D. J. & Cooper, A. I. (2012). *Angew. Chem. Int. Ed.* **51**, 7154–7157. 10.1002/anie.201202849 22684980

[bb24] Hurle, K., Lubauer, J., Belli, R. & Lohbauer, U. (2022). *Dent. Mater.* **38**, 1558–1563. 10.1016/j.dental.2022.07.009 35927096

[bb61] Karki, S., Friščić, T. & Jones, W. (2009). *CrystEngComm* **11**, 470–481.

[bb25] Kassouf, N., Zappi, A., Monticelli, M. & Melucci, D. (2024). *Chemosensors* **12**, 227.

[bb26] Kitaigorodsky, A. I. (1984). *Mixed crystals.* Berlin, Heidelberg: Springer.

[bb27] Kobayashi, T., Sato, Y., Tonna, R., Matsumura, D., Sasaki, T. & Ikeda-Ohno, A. (2024). *Dalton Trans.* **53**, 18616–18628. 10.1039/d4dt02272f 39474922

[bb28] le Roex, T., Nassimbeni, L. R. & Weber, E. (2007). *Chem. Commun.* p. 1124. 10.1039/b618782j 17347713

[bb29] Lopresti, M., Mangolini, B., Conterosito, E., Milanesio, M. & Palin, L. (2023). *Cryst. Growth Des.* **23**, 1389–1402.

[bb30] Lusi, M. (2018). *Cryst. Growth Des.* **18**, 3704–3712.

[bb31] Lusi, M., Vitorica-Yrezabal, I. J. & Zaworotko, M. J. (2015). *Cryst. Growth Des.* **15**, 4098–4103.

[bb32] Macchietti, L., Casali, L., Emmerling, F., Braga, D. & Grepioni, F. (2024). *RSC Mechanochem.* **1**, 106–115.

[bb65] Macrae, C. F., Sovago, I., Cottrell, S. J., Galek, P. T. A., McCabe, P., Pidcock, E., Platings, M., Shields, G. P., Stevens, J. S., Towler, M. & Wood, P. A. (2020). *J. Appl. Cryst.* **53**, 226–235. 10.1107/S1600576719014092 PMC6998782 32047413

[bb33] Narala, S., Nyavanandi, D., Srinivasan, P., Mandati, P., Bandari, S. & Repka, M. A. (2021). *J. Drug. Deliv. Sci. Technol.* **61**, 102209. 10.1016/j.jddst.2020.102209 PMC7946067 33717230

[bb34] Newsome, W. J., Ayad, S., Cordova, J., Reinheimer, E. W., Campiglia, A. D., Harper, J. K., Hanson, K. & Uribe-Romo, F. J. (2019). *J. Am. Chem. Soc.* **141**, 11298–11303. 10.1021/jacs.9b05191 31265284

[bb35] Oliveira, M. A., Peterson, M. L. & Klein, D. (2008). *Cryst. Growth Des.* **8**, 4487–4493.

[bb62] Orola, L. & Veidis, M. V. (2009). *CrystEngComm* **11**, 415.

[bb36] Parrish, W. & Langford, J. I. (2006). *International tables for crystallography*, Vol. C, edited by E. Prince, pp. 48–49. Chester: International Union of Crystallography.

[bb37] Pedregosa, F., Varoquaux, G., Gramfort, A., Michel, V., Thirion, B., Grisel, O., Blondel, M., Prettenhofer, P., Weiss, R., Dubourg, V., Vanderplas, J., Passos, A., Cournapeau, D., Brucher, M., Perrot, M. & Duchesnay, É. (2011). *J. Mach. Learn. Res.* **12**, 2825–2830.

[bb38] Rajalahti, T. & Kvalheim, O. M. (2011). *Int. J. Pharm.* **417**, 280–290. 10.1016/j.ijpharm.2011.02.019 21335075

[bb39] Romasanta, A. K. S., Braga, D., Duarte, M. T. & Grepioni, F. (2017). *CrystEngComm* **19**, 653–660.

[bb40] Ryan, C. G., Clayton, E., Griffin, W. L., Sie, S. H. & Cousens, D. R. (1988). *Nucl. Instrum. Methods Phys. Res. B* **34**, 396–402.

[bb41] Shaikh, R., O’Brien, D. P., Croker, D. M. & Walker, G. M. (2018). *Computer aided chemical engineering*, Vol. 41, *Process systems engineering for pharmaceutical manufacturing*, edited by R. Singh & Z. Yuan, pp. 27–65. Elsevier.

[bb42] Shemchuk, O., Braga, D. & Grepioni, F. (2016). *Chem. Commun.* **52**, 11815–11818. 10.1039/c6cc06615a 27722256

[bb43] Skou, P. B., Berg, T. A., Aunsbjerg, S. D., Thaysen, D., Rasmussen, M. A. & van den Berg, F. (2017). *Appl. Spectrosc.* **71**, 410–421. 10.1177/0003702816654165 27899431

[bb44] Spoletti, E., Verma, V., Cappuccino, C. & Lusi, M. (2023). *Chem. Commun.* **59**, 14321–14324. 10.1039/d3cc04725c 37971413

[bb45] Srisanga, S. & ter Horst, J. H. (2010). *Cryst. Growth Des.* **10**, 1808–1812.

[bb46] Tao, Y., Gao, Y., Zhang, B., Hu, K., Xie, Y., Zhang, L., Yang, S. & Lu, Y. (2025). *Crystals* **15**, 38.

[bb60] Thompson, L. J., Voguri, R. S., Cowell, A., Male, L. & Tremayne, M. (2010). *Acta Cryst.* C**66**, o421–o424. 10.1107/S0108270110027319 20679721

[bb47] Urbano-Cuadrado, M., Ruiz, I. L. & Gómez-Nieto, M. Á. (2008). *Int. J. Comput. Math.* **85**, 691–702.

[bb48] Virtanen, P., Gommers, R., Oliphant, T. E., Haberland, M., Reddy, T., Cournapeau, D., Burovski, E., Peterson, P., Weckesser, W., Bright, J., van der Walt, S. J., Brett, M., Wilson, J., Millman, K. J., Mayorov, N., Nelson, A. R. J., Jones, E., Kern, R., Larson, E., Carey, C. J., Polat, İ., Feng, Y., Moore, E. W., VanderPlas, J., Laxalde, D., Perktold, J., Cimrman, R., Henriksen, I., Quintero, E. A., Harris, C. R., Archibald, A. M., Ribeiro, A. H., Pedregosa, F., van Mulbregt, P., Vijaykumar, A., Bardelli, A. P., Rothberg, A., Hilboll, A., Kloeckner, A., Scopatz, A., Lee, A., Rokem, A., Woods, C. N., Fulton, C., Masson, C., Häggström, C., Fitzgerald, C., Nicholson, D. A., Hagen, D. R., Pasechnik, D. V., Olivetti, E., Martin, E., Wieser, E., Silva, F., Lenders, F., Wilhelm, F., Young, G., Price, G. A., Ingold, G., Allen, G. E., Lee, G. R., Audren, H., Probst, I., Dietrich, J. P., Silterra, J., Webber, J. T., Slavič, J., Nothman, J., Buchner, J., Kulick, J., Schönberger, J. L., de Miranda Cardoso, J. V., Reimer, J., Harrington, J., Rodríguez, J. L. C., Nunez-Iglesias, J., Kuczynski, J., Tritz, K., Thoma, M., Newville, M., Kümmerer, M., Bolingbroke, M., Tartre, M., Pak, M., Smith, N. J., Nowaczyk, N., Shebanov, N., Pavlyk, O., Brodtkorb, P. A., Lee, P., McGibbon, R. T., Feldbauer, R., Lewis, S., Tygier, S., Sievert, S., Vigna, S., Peterson, S., More, S., Pudlik, T., Oshima, T., Pingel, T. J., Robitaille, T. P., Spura, T., Jones, T. R., Cera, T., Leslie, T., Zito, T., Krauss, T., Upadhyay, U., Halchenko, Y. O. & Vázquez-Baeza, Y. (2020). *Nat. Methods* **17**, 261–272.

[bb49] Vogrin, J., Hodge, H., Santini, T., Peng, H. & Vaughan, J. (2019). *Light metals*, edited by C. Chesonis, pp. 93–99. Cham: Springer Inter­national Publishing.

[bb50] Wiklund, S., Nilsson, D., Eriksson, L., Sjöström, M., Wold, S. & Faber, K. (2007). *J. Chemometr.* **21**, 427–439.

[bb51] Wold, S., Sjöström, M. & Eriksson, L. (2001). *Chemometr. Intell. Lab. Syst.* **58**, 109–130.

[bb52] Wouters, J., Braga, D., Grepioni, F., Aerts, L. & Quéré, L. (2018). *Polymorphism in the pharmaceutical industry*, edited by R. Hilfiker & M. V. Raumer, pp. 61–90. Wiley.

[bb53] Xiong, Y., Zhao, Z., Zhao, W., Ma, H., Peng, Q., He, Z., Zhang, X., Chen, Y., He, X., Lam, J. W. Y. & Tang, B. Z. (2018). *Angew. Chem. Int. Ed.* **57**, 7997–8001. 10.1002/anie.201800834 29736955

[bb54] Xu, Z., Hean, D., Yuan, J. & Wolf, M. O. (2022). *Chem. Sci.* **13**, 6882–6887. 10.1039/d2sc01922a PMC9200050 35774161

[bb55] Ying, P., Yu, J. & Su, W. (2021). *Adv. Synth. Catal.* **363**, 1246–1271.

[bb56] Yuan, Z., Li, Z., Luo, J., Nawaz, A., Zhang, B. & Dessie, W. (2024). *Molecules* **29**, 2379. 10.3390/molecules29102379 PMC11123969 38792239

[bb57] Zappi, A., Maini, L., Galimberti, G., Caliandro, R. & Melucci, D. (2019). *Eur. J. Pharm. Sci.* **130**, 36–43. 10.1016/j.ejps.2019.01.014 30654113

[bb58] Zhang, L. & Garcia-Munoz, S. (2009). *Chemometr. Intell. Lab. Syst.* **97**, 152–158.

[bb59] Zhen, Y., Tanaka, H., Harano, K., Okada, S., Matsuo, Y. & Nakamura, E. (2015). *J. Am. Chem. Soc.* **137**, 2247–2252. 10.1021/ja513045a 25626088

