# Supplementary material for: Solid solution quantification from full powder X-ray diffraction profile: novel application of multivariate calibration
Source: J Appl Crystallogr. 2025 Sep 4;58(Pt 5):1700–11. doi: 10.1107/S1600576725006910 (PMC12502876; doi:10.1107/S1600576725006910)
Supplement: Supplementary file 1 [file j-58-01700-sup1.pdf]

JOURNAL OF
APPLIED
CRYSTALLOGRAPHY

Volume 58 (2025)

Supporting information for article:

Solid solution quantification from full powder X-ray diffraction profile: novel application of multivariate calibration

Laura Macchietti, Nicholas Kassouf, Giovanni Valenti, Dora Melucci and Fabrizia Grepioni

S1. Analysis of the peak positions and relative shifts of the NA-SS experimental data

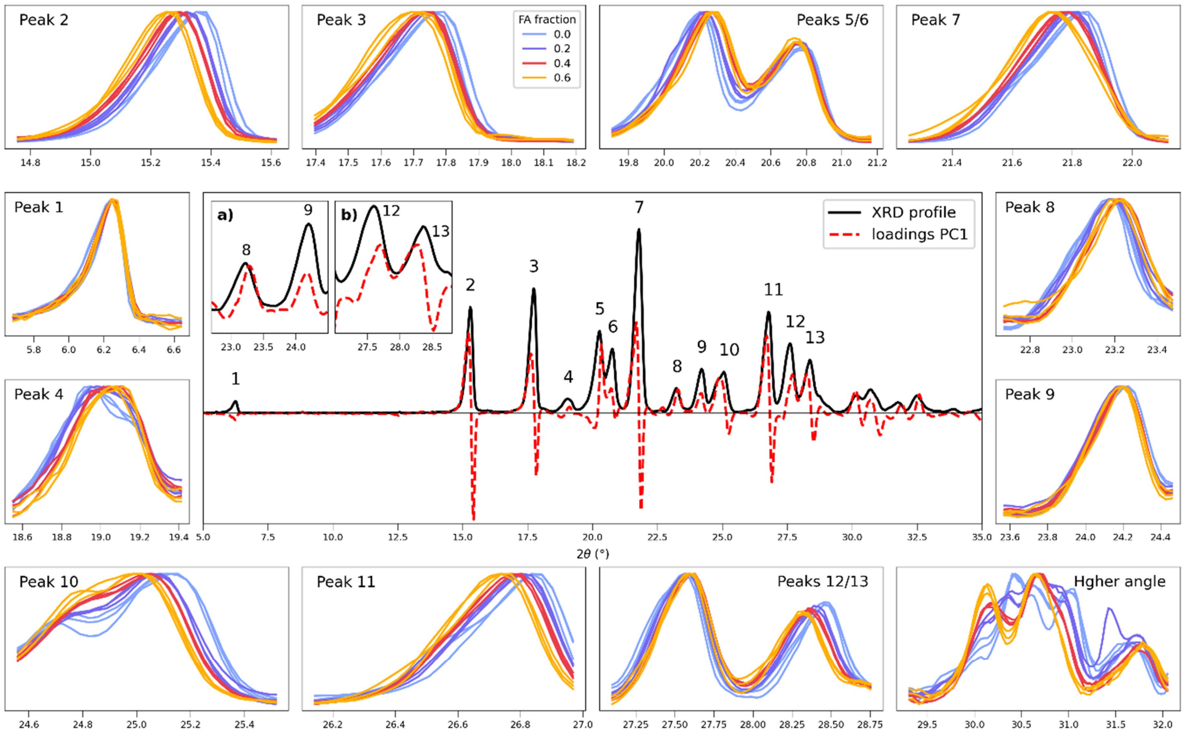

Figure S1 Central plot: comparison of the experimental profile of $NA_2{\cdot}FA_xSA_{(1-x)}$ solid solution (solid line) and the PC1 loadings (dotted line) from PCA analysis of the calibration dataset. Numbers mark the peaks considered for the statistical analysis in Table S1. Enlargements of the 2θ range from 23 to 29° are included in panels a) and b). External plots: details of the experimental XRD data from the calibration dataset (FA= 0, 0.2, 0.4, 0.6) after manual alignment, matching the numbered peaks in the full profile. For each plot, a normalization between 0 and 1 was applied to the selected range, to allow for a better visualization of the progression.

The first thirteen peaks of the $NA_2{\cdot}FA_xSA_{(1-x)}$ profile (Fig. S1) were analysed to study the magnitude of the shift induced by the substitution and its experimental variability. The aligned calibration set, composed of three samples for each solid solution composition (FA = 0, 0.2, 0.4, 0.6), was considered for statistical analysis. In Table S1 the average 2θ value of each peak (Table S1a) and the standard deviation associated to the three replicates (Table S1b) are reported, for all the compositions considered. The goodness of the alignment and the reproducibility of the technique is confirmed by the standard deviation of the position, affecting the third decimal digit, with only the 0.6/0.4 (FA/SA) composition showing higher deviation on average (0.01°); this greater oscillation of the profile, noticed for the samples with the maximum substitution, is consistent with the limit of miscibility and

the observed instability of the composition. Calculated from the mean position values, the absolute shift between the two limit compositions ($|\Delta 2\theta_{0/0.6}|$ Table S1a) are compared in Fig. S2, where it can be observed how the peaks are differently affected by the cell expansion: in addition to the reference peak, used for the alignment (peak 1), four more peaks (peaks 6, 8, 9 and 12) present a maximum shift below average. The $\Delta 2\theta$ of peak 9 in particular is comparable with the experimental variability and can be considered not affected by the shift, while the others have a minimal variation; confirmation from the experimental data can be found on the respective peak detail in Fig. S1. Moreover, looking at the loadings profile from the PCA model (red dotted line in the central plot, and detail in Fig. S1a/b), the absence or a minimal split of the profile is evident at the peaks associated with low $\Delta 2\theta$, supporting how the peak split behaviour is characteristic of the solid solution progression and how the PCA analysis, and in particular the loadings plot, can easily reveal the main features of the XRD profile.

Table S1 a) Mean peak position calculated for the numbered peaks in Fig. S1 of each solid solution composition (three samples per FA fraction). In the last column the absolute value $|\Delta 2\theta_{0/0.6}|$ of the difference between fraction 0 and 0.6 is reported, corresponding to the shift induced by the maximum substitution. b) Standard deviations associated with the three samples evaluated for each composition.

	a) Average peak position at different FA fraction					b) Standard deviation of peak position			
**Peak #**	**0**	**0.2**	**0.4**	**0.6**	$\mathbf{|\Delta 2\theta_{0/0.6}|}$	**0**	**0.2**	**0.4**	**0.6**
1	6.241	6.242	6.243	6.242	0.001	0.004	0.002	0.004	0.002
2	15.350	15.313	15.287	15.253	0.10	0.005	0.003	0.005	0.009
3	17.762	17.735	17.726	17.70	0.06	0.005	0.005	0.006	0.01
4	18.96	19.018	19.044	19.06	0.10	0.01	0.007	0.004	0.01
5	20.194	20.220	20.256	20.27	0.07	0.009	0.005	0.007	0.01
6	20.771	20.750	20.749	20.74	0.03	0.006	0.009	0.007	0.01
7	21.822	21.791	21.772	21.733	0.09	0.007	0.005	0.006	0.005
8	23.168	23.183	23.222	23.21	0.05	0.003	0.002	0.004	0.01
9	24.200	24.185	24.190	24.18	0.01	0.007	0.008	0.006	0.01
10	25.114	25.059	25.027	24.98	0.14	0.005	0.006	0.009	0.02
11	26.832	26.795	26.769	26.732	0.10	0.005	0.005	0.005	0.009
12	27.549	27.567	27.588	27.585	0.04	0.005	0.006	0.007	0.009
13	28.454	28.390	28.356	28.316	0.14	0.003	0.006	0.004	0.007

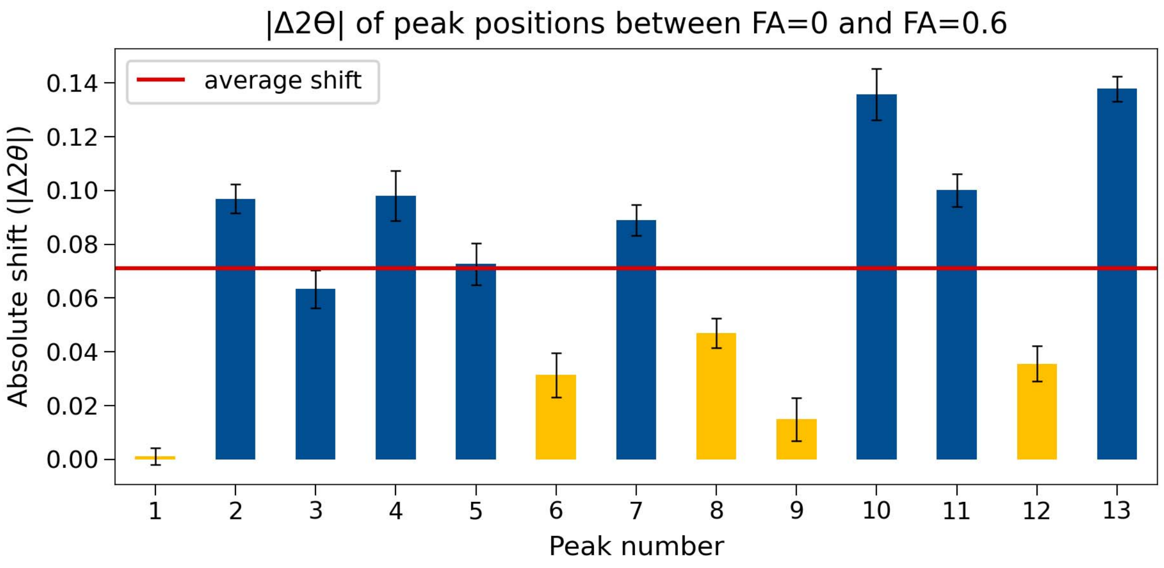

Figure S2 Bar plot showing the $\Delta 2\theta_{0/0.6}$ shift (Table 1a) for the thirteen peaks of the **NA-SS** profile. The red line marks the average value; the peaks significantly below average are marked in yellow. Error bars correspond to the cumulative standard deviation across all fractions for the respective peak.

S2. Optimization of the auto-alignment algorithm

As described in Section 3.1.4, the automated alignment procedure required the definition of a method for the correct identification of the peak position. By default the function used for peak search (Section 2.5.1) returns the point of maximum intensity, which is particularly sensitive to the peak shape and the sampling step; as evident from the detail of the reference peak in Fig. S3a where the result of the identification is reported, showing the first sample used as reference for the alignment (red) and a subsequent sample (S10 – black). The peak marked with the blue X is found shifted to the left for the reference profile and to the right for the S10 sample, causing an imperfect alignment (Fig. S3b).

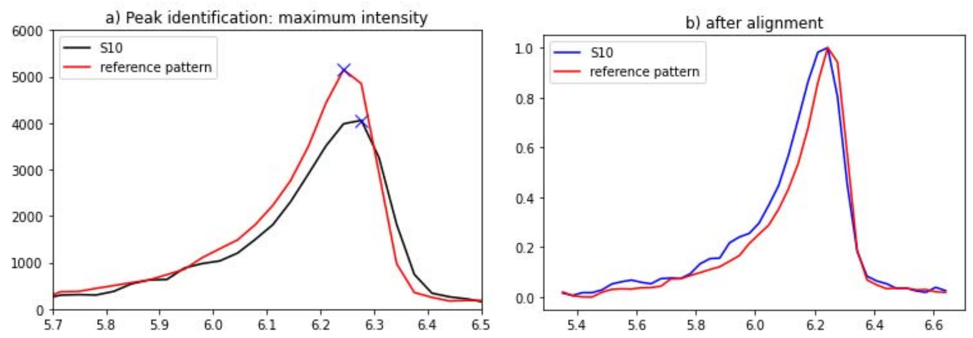

Figure S3 a) Detail of the alignment peak of the **NA-SS** dataset, showing the identification of the peak position as the maximum point of intensity of the profile (blue X). The original experimental data of the first sample of the dataset, considered as reference for the alignment (red line) and a following calibration sample named S10 (black) are reported. b) Result of the alignment according to the identification of the peak in panel a). The S10 sample profile after the alignment is reported in blue, with the profiles normalized between 0-1 for a better visualization of the alignment.

To determine the centre of the peak more correctly, the middle point of the peak width was considered and in Fig. S4b the result of the alignment for the S10 sample is reported, showing a correct alignment of the peaks after applying this second procedure. As first attempt, the common width at half height was considered (Fig. S4a), that appeared satisfactory for the S10 sample, but in presence of high peak asimmetry, as in the case of sample S18 (Fig. S5a), this methodology also was affected by error. As shown in Fig. S5a, if assymetry is present the width middle point appears shifted towards the broader side instead of the centre of the peak, introducing an error in the procedure. Considering the width closer to the maximum helped avoid the problem, and for the **NA-SS** dataset the width at 80% of the height was found satisfactory (Fig. S5c) for an alignment equivalent to the manual procedure.

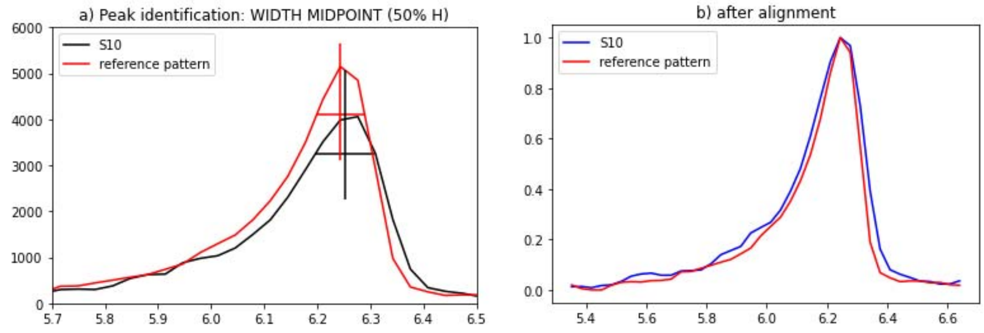

Figure S4 a) Detail of the alignment peak of the **NA-SS** dataset, showing the identification of the peak position as the central point of the width at 50% of the peak height. The original experimental data of the first sample of the dataset, considered as reference for the alignment (red line) and a following calibration sample named S10 (black) are reported. b) Result of the alignment according to the identification of the peak in panel a). The S10 sample profile after the alignment is reported in blue, with the profiles normalized between 0-1 for a better visualization of the alignment.

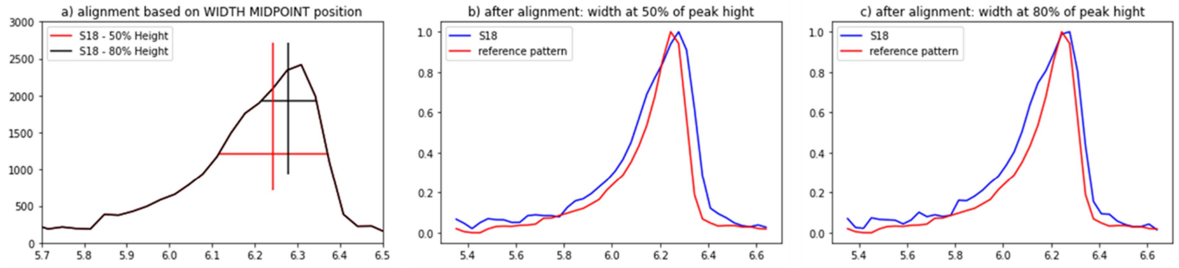

Figure S5 a) Detail of the alignment peak of the **NA-SS** dataset for the calibration sample S18, comparing the identification of the peak position as the central point of the width at 50% (red) and 80% (black) of the peak height. Results of the alignment according to the two identification procedures in panel a) are shown in panels b) and c) respectively. The profiles are plotted normalized between 0-1 for a better visualization of the alignment.

S3. Additional XRD data for the IN-SS system

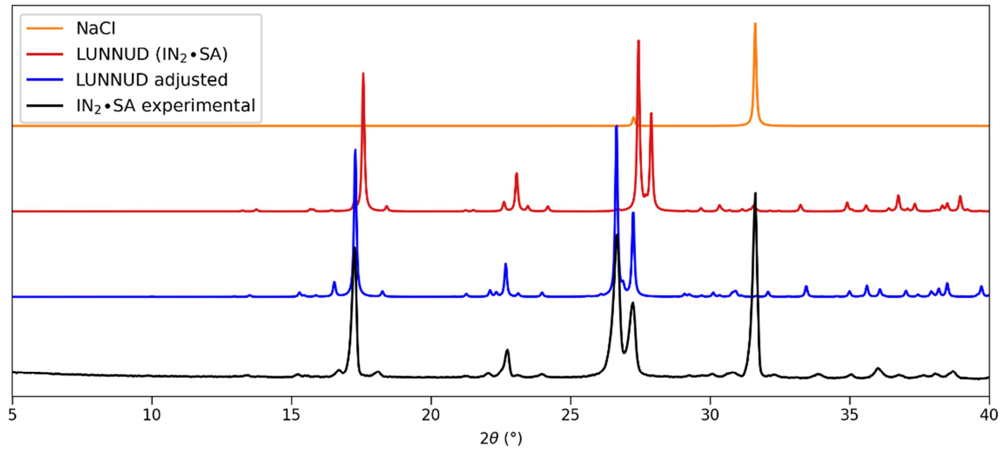

Figure S6 Comparison of experimental pattern of IN2·SA (bottom line) with deposited reference data at low temperature (refcode LUNNUD), second line from the top, and the profile optimized with the autoFidel function (Mercury – CCDC), third line from the top. No change is evident in the adjusted profile except for the thermal expansion correction, being therefore a valid reference for the experimental XRD; the extra peak at 31.6° 2θ, matches the NaCl internal standard (top line).

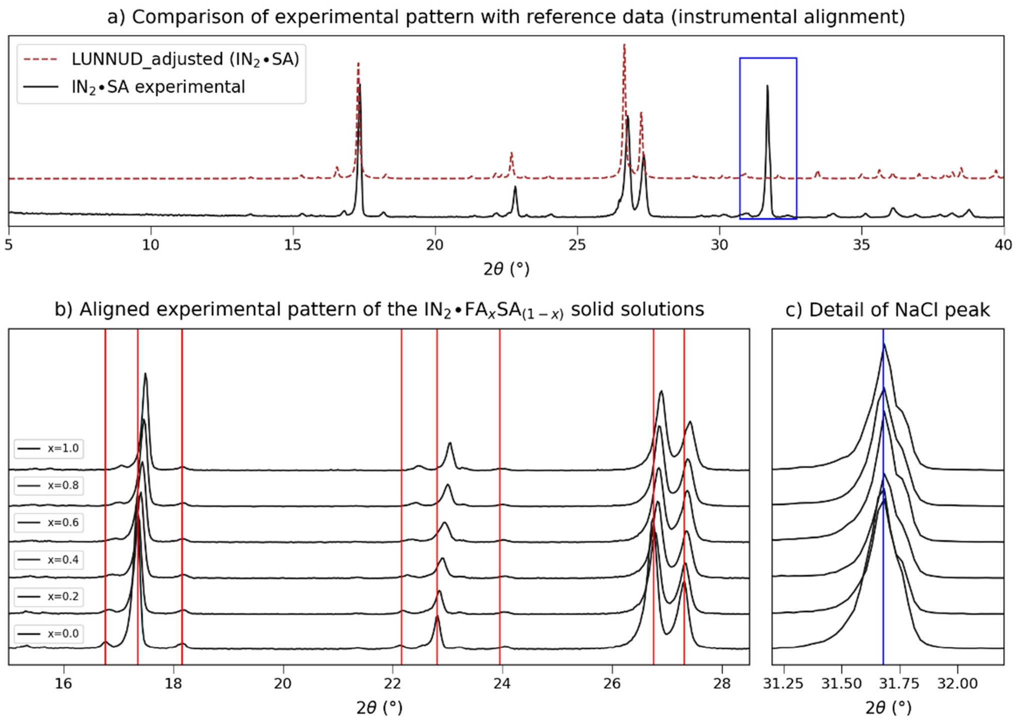

Figure S7 Experimental PXRD data of the **IN-SS** dataset after instrumental alignment: a) comparison of the experimental profile (solid line) with the adjusted (see Fig. S6) calculated pattern from single crystal (dotted line). b) Detail of the range 15 to 28°2θ, of the experimental patterns of solid solutions with increasing content of FA (x). The vertical red lines help trace the shift affected by the peak positions. c) Detail of the NaCl peak.

S4. Additional plots from PCA calculations

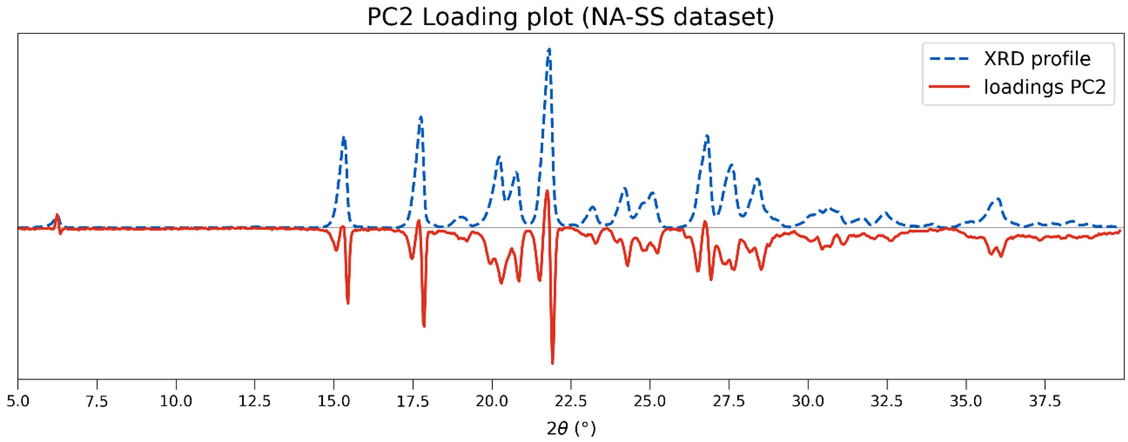

Figure S8 PC2 loadings plot (red solid line) from exploratory PCA analysis of the full **NA-SS** dataset. For better interpretation of the role of the original variables within the model, the experimental XRD pattern is also reported in the plot (blue dotted line).

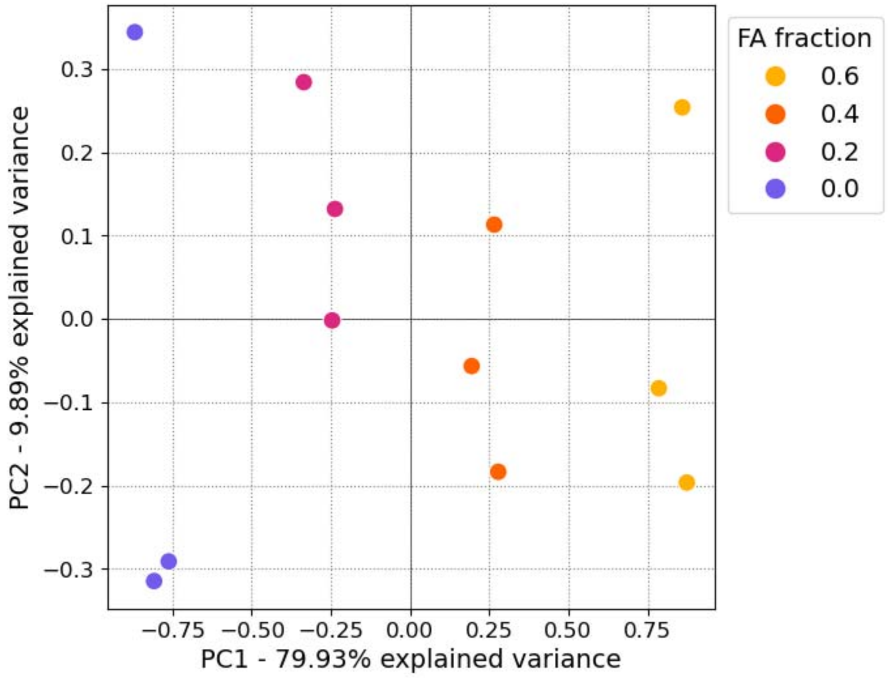

Figure S9 Scores plot (PC1 vs. PC2) of **NA-SS** calibration dataset. Samples separate along PC1 direction according to the solid solution composition (FA fraction) marked by colours according to the legend.

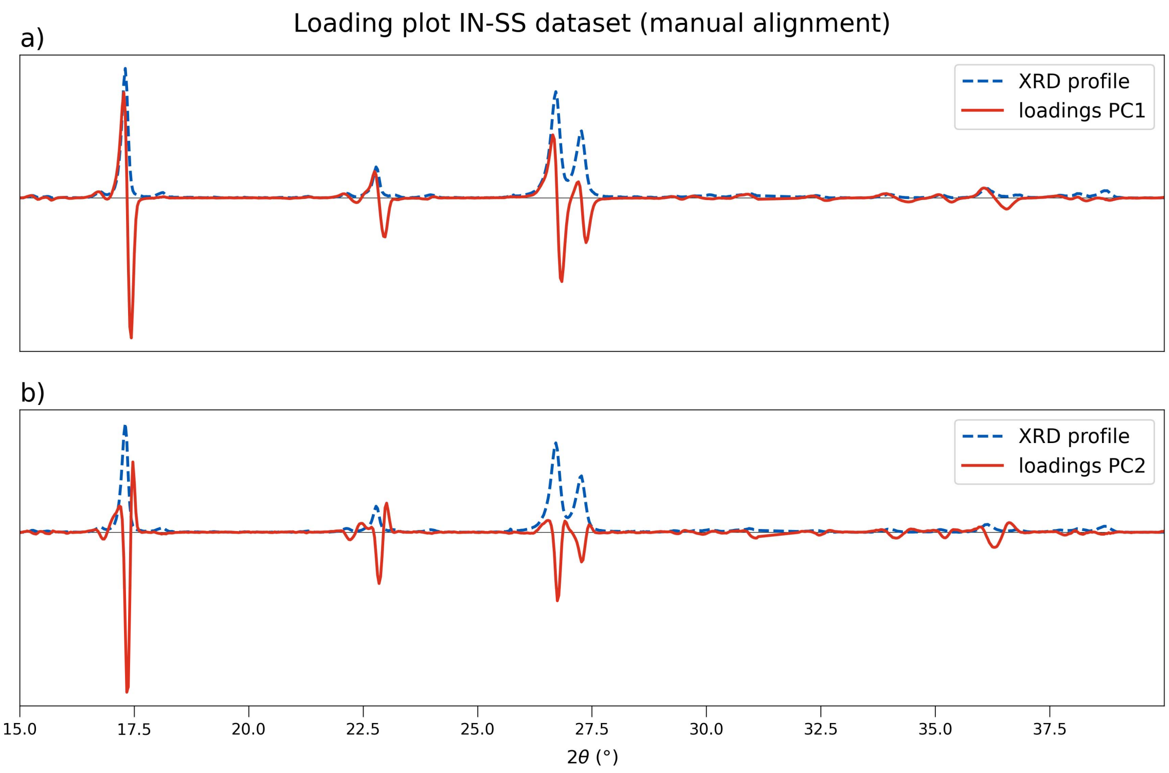

Figure S10 Loadings plot (red solid line) of the PC1 component (a) and the PC2 component (b), from PCA analysis of the **IN-SS** calibration dataset (manual alignment). In both figures loadings are compared with the experimental XRD profile (blue dotted line).

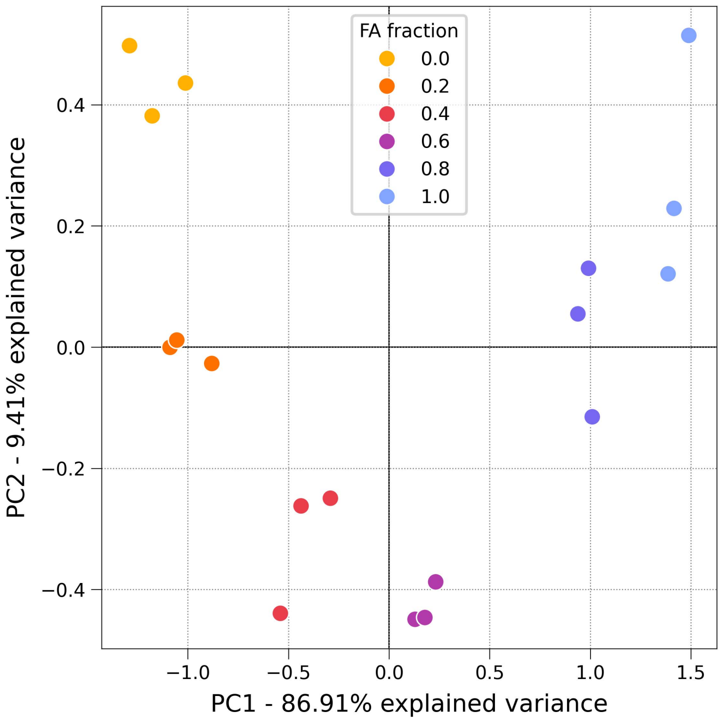

Figure S11 Scores plot (PC1 vs. PC2) of the instrumentally aligned **IN-SS** calibration data, showing similar separation of the samples as in the case of the manual alignment dataset (Fig. 12).

S5. Additional data used for PLS model selection

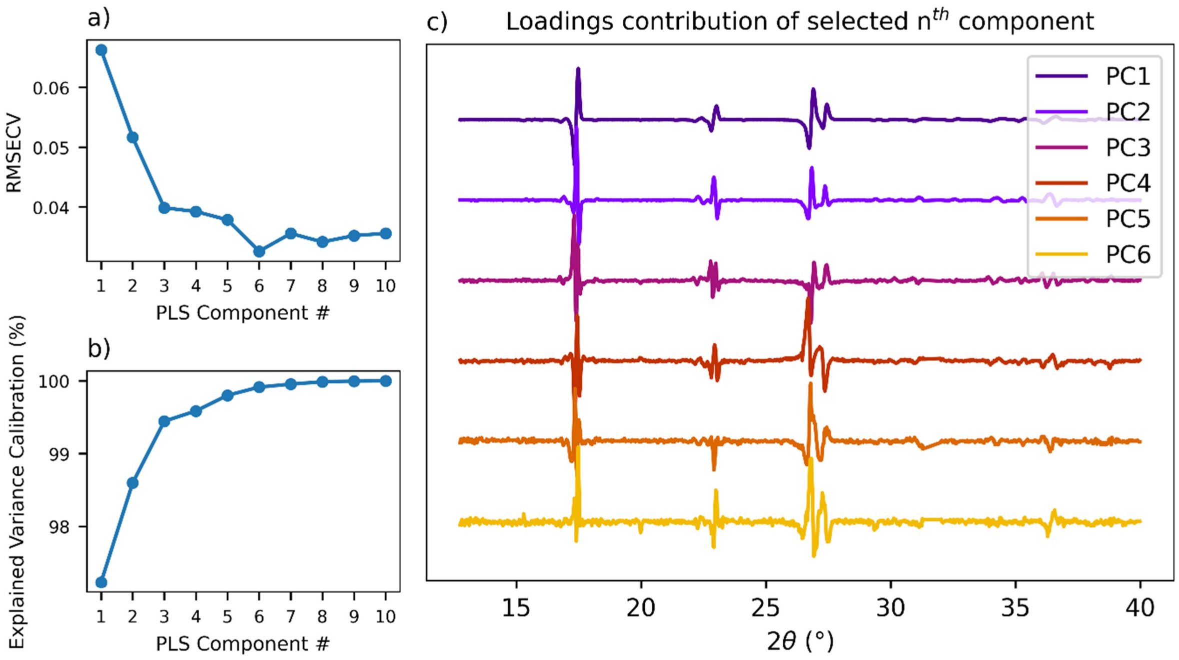

Figure S12 Additional features of the PLS models used for components selection, relative to the **IN-SS** calibration dataset (instrumental alignment): a) RMSECV as function of the number of components; b) explained variance associated to the first 10 components; c) loadings plot of the first six components, selected as the optimum value for the building of the model.

S6. Pawley refinement results of the NA-SS system

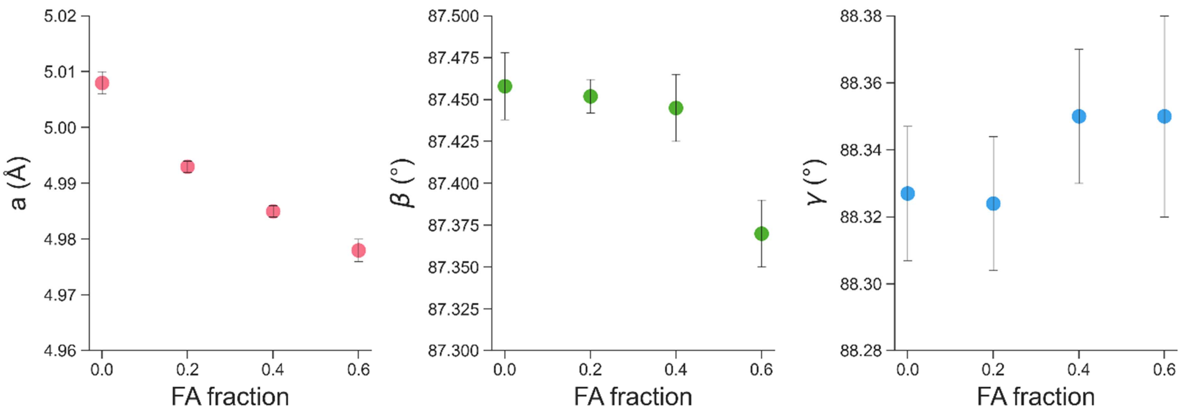

Figure S13 Plots of selected unit cell parameters (a lengths, β and γ angle) resulting from Pawley analyses of PXRD profiles, collected for the $NA_2{\cdot}FA_xSA_{(1-x)}$ solid solution at different values of the FA molar fraction x.

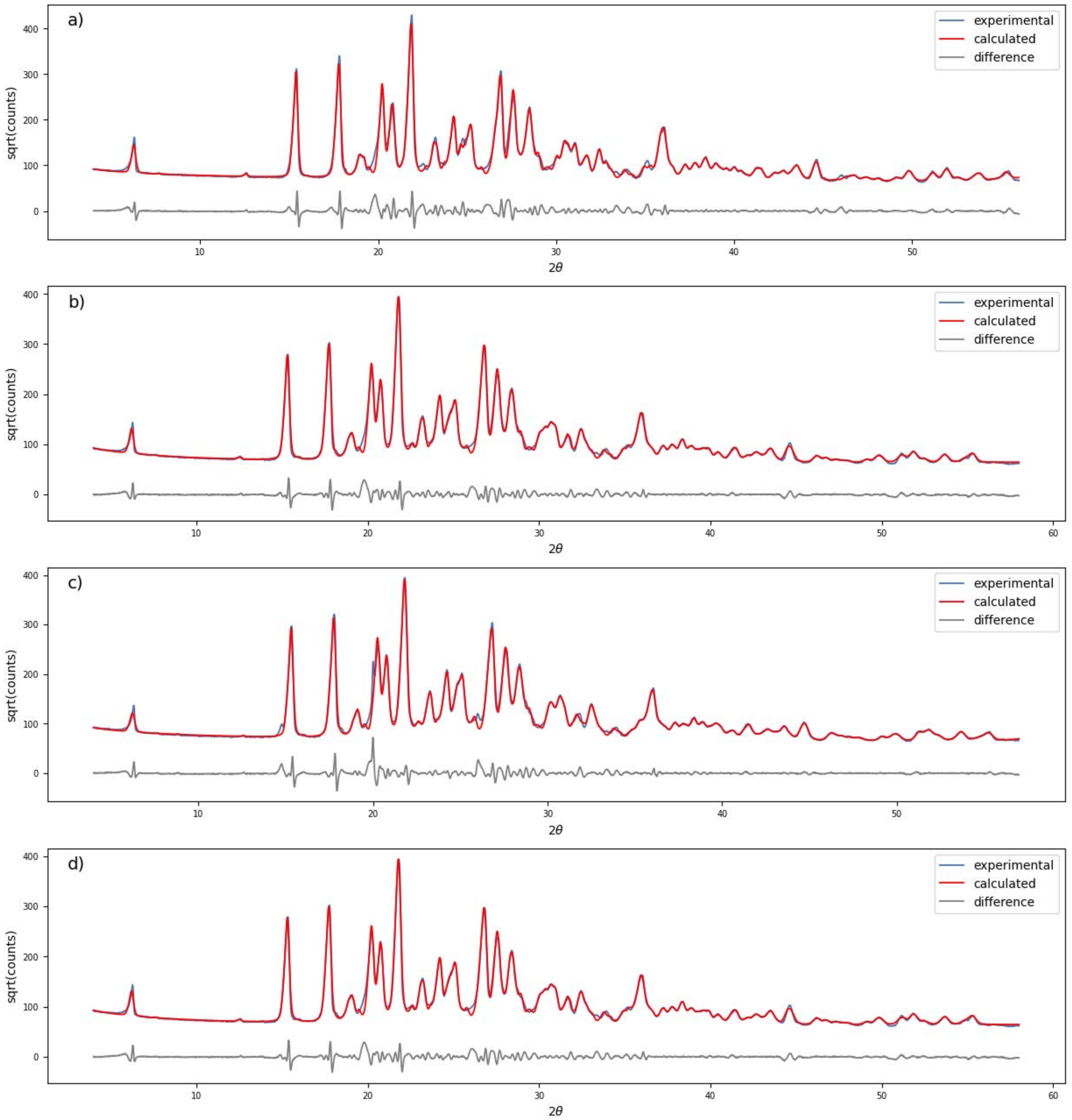

Figure S14 Results from Pawley fitting. Experimental (blue), calculated (red) and difference (grey) patterns for $NA_2{\cdot}FA_xSA_{(1-x)}$ with: a) x=0, b) x=0.2, c) x=0.4, d) x=0.6.
